# From Fish Waste to Stable Omega‐3 Bioactive Lipids: Encapsulation Strategies and a Conceptual Framework for Antioxidant and Antimicrobial Functional Food Fortification

**DOI:** 10.1002/fsn3.72178

**Published:** 2026-08-04

**Authors:** Abebe Desalegn, Habtamu Admassu, Gadissa Mosisa, Gebeyehu Ayele, Molla Abeje, Tanaji Kudre

**Affiliations:** ^1^ Department of Chemical Engineering, Faculty of Technology Wollega University Shambu Campus Shambu Ethiopia; ^2^ Food Process Engineering Program, Department of Chemical Engineering, College of Engineering Addis Ababa Science and Technology University Addis Ababa Ethiopia; ^3^ Biotechnology and Bioprocess Center of Excellence Addis Ababa Science and Technology University Addis Ababa Ethiopia; ^4^ Department of Food Engineering, College of Engineering and Technology Dilla University Dilla Ethiopia; ^5^ Department of Food Engineering, College of Engineering and Technology Wolkite University Wolkite Ethiopia; ^6^ Meat and Marine Science Department CSIR‐ Central Food Technological Research Institute Mysore India

**Keywords:** antimicrobial, antioxidant, encapsulation, fish oil, fortification

## Abstract

Valorization of fish processing waste into high‐value omega‐3 bioactive lipids represents a sustainable strategy for the development of functional foods. However, the incorporation of long‐chain polyunsaturated fatty acids (PUFAs), such as EPA and DHA, is constrained by oxidative instability, off‐flavor formation, and poor water solubility. This review critically integrates advanced encapsulation technologies with mechanistic insights into their roles in enhancing lipid stability, modulating gastrointestinal release, and improving bioavailability within diverse food matrices. Encapsulation systems not only protect PUFAs during processing and storage but also enable controlled release, thereby enhancing physiological uptake. In addition, fish oil‐derived lipids exhibit antioxidant properties through modulation of oxidative pathways and antimicrobial effects through membrane disruption and metabolic interference. A conceptual framework for the design of fortified foods is proposed, emphasizing controlled release, synergistic interactions with food components, and sustained biological functionality. By linking mechanistic understanding with practical formulation strategies, this review provides a comprehensive roadmap for the effective and safe utilization of fish waste‐derived omega‐3 lipids in next‐innovative functional foods.

## Graphical abstract

Fish waste is valorized into omega‐3‐rich bioactive lipids (EPA and DHA), followed by stabilization through advanced encapsulation technologies to prevent oxidation and improve storage stability. Encapsulation protects polyunsaturated fatty acids from oxidation, improves storage stability, enables controlled gastrointestinal release, and enhances bioavailability. The encapsulated lipids exhibit antioxidant and antimicrobial activities, facilitating their incorporation into functional food matrices such as bakery products, dairy products, and meat products. This integrated approach provides a sustainable pathway for converting fish waste into stable, bioactive ingredients for next‐generation functional foods with enhanced health benefits.

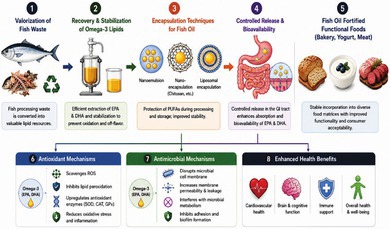

## 1 Introduction

Fish processing has experienced a significant global expansion, resulting in substantial amounts of byproduct biomass, with up to 20%–80% of the harvested fish going to waste (Coppola et al. 2021). These wastes contain valuable nutritionally active compounds, including long‐chain omega‐3 polyunsaturated fatty acids (PUFAs) such as Eicosapentaenoic acid (EPA) and docosahexaenoic acid (DHA), which are essential for human health, especially associated with reduced cardiovascular risks and chronic inflammation (Debbarma et al. 2023). These wastes hold great potential for conversion into high‐value resources for circular bioeconomies, which otherwise contribute to environmental burdens and the loss of functional food potential (Korma et al. 2025). The main challenges of these bioactive PUFAs are highly prone to oxidation and sensory deterioration that could limit their direct incorporation into foods. For thus, effective stabilization strategies are necessary to maintain bioactivity during processing (Rahim et al. 2025).

Encapsulation technologies are needed not only to enhance the physicochemical stability and oxidative resistance of fish oil but also to provide controlled release profiles and sensory masking to meet the requirements for consumer‐acceptable functional food formulations (Rahim et al. 2025). Micro/nanoencapsulation technologies such as emulsions, nanostructured lipid carriers, and gel matrices have been employed to shield omega‐3 lipids from environmental stressors, enhance their bioaccessibility, and modify lipid release kinetics during gastrointestinal transit (Venugopalan et al. 2021). Optimizing these techniques, such as the selection of wall material, matrix structure formation, and natural antioxidant integration, is being identified as a key factor in extending shelf life and the functional efficacy of fortified foods (Perez‐Palacios et al. 2022).

In functional food design, controlled release mechanisms were essential for PUFA targeted delivery to facilitate synergistic interactions with other food components to promote nutrient uptake, alter digestion behavior, and provide sustained biological activity (Venugopalan et al. 2021). Controlled delivery frameworks help encapsulated fish oil resist processing stresses while maintaining endogenous free radical scavenging benefits that minimize oxidative damage in food matrices and human tissues (Honrado et al. 2022). Moreover, long‐chain omega‐3s have antimicrobial activity through membrane disruption, energy metabolism, key enzyme inhibition, and reduction of biofilm formation, thereby providing the potential to inhibit spoilage and pathogenic microbes in fortified foods (Jimenez‐Champi et al. 2024). The dual antioxidant (Aldhafiri 2022) and antimicrobial properties of omega‐3s make them a promising candidate for formulation of functional foods.

This review provides a comprehensive overview of the sustainable valorization of fish processing waste for the recovery of bioactive lipids, with a particular focus on omega‐3‐rich fish oil. It critically evaluates advanced encapsulation strategies aimed at enhancing oxidative stability, enabling controlled release, and improving the bioavailability of fish oil in food systems. Furthermore, the review examines the functional implications of fish oil fortification, including its potential antioxidant and antimicrobial effects in diverse functional food applications. Ultimately, this work seeks to develop a conceptual framework to guide future research and support the design of stable, effective, and innovative fish oil‐enriched functional foods.

## 2 Materials and Methods

The present paper reviews the recent findings on the conversion of fish processing byproducts into stable fish oil and its utilization to supplement functional foods. Articles published from 2020 to 2025 in Scopus, Web of Science, PubMed, Science Direct, and Springer Link were screened. The keywords used to perform the searches were fish waste valorization, omega‐3, encapsulation, antioxidant and antimicrobial activities, and functional food fortification. We only included peer‐reviewed studies that were quality reports by carefully evaluating the relevance before being included. The relevant studies were categorized as follows: (i) valorization of fish waste for bioactive lipid recovery; (ii) encapsulation techniques for fish oil; (iii) controlled release of fish oil; (iv) antioxidant and antimicrobial functionality of fish oil‐fortified foods; and (v) fish oil‐fortified functional foods. The studies were reviewed for potential antioxidant and antimicrobial capabilities, their scalability for production, and applicability to food systems to bring the evidence together to develop a pathway linking fish waste byproducts to encapsulated fish oil fortified in functional food.

## 3 Valorization of Fish Waste for Bioactive Lipid Recovery

Fish waste generated during harvesting and industrial fish processing accounts for approximately 20%–80% of total fish biomass, depending on species, filleting efficiency, and processing methods, with major fractions including heads (9%–12%), viscera (12%–18%), bones and frames (9%–15%), skin and fins (1%–3%), and scales (2%–7%) (Coppola et al. 2021; Kumar et al. 2025). Those wastes are rich in valuable biomolecules, containing approximately 15%–60% proteins, 20%–40% lipids, 1%–5% minerals, and substantial quantities of collagen, gelatin, enzymes, peptides, and omega‐3 fatty acids (Caruso et al. 2020). Fish viscera generally contain 20%–50% lipids, while fish liver may contain 30%–60% lipids, making them the principal sources of recoverable fish oils and omega‐3 fatty acids. Fish heads and frames commonly contain 10%–20% lipids, whereas skin contains approximately 5%–10% lipids depending on species (Waqar et al. 2025). The total fish by‐product oils usually contain 2%–30% extractable fats, with polyunsaturated fatty acids (PUFAs) representing 6.08%–33.77% of total fatty acids. EPA and DHA are the dominant bioactive omega‐3 fatty acids in fish waste lipids, commonly accounting for 5%–26% of total fatty acids in recovered fish oils (Jimenez‐Champi et al. 2024). In trout processing by‐products, EPA contents reached 8.7%, 7.9%, and 6.4% in spines, heads, and viscera, respectively, while DHA contents ranged from 6.3%, 6.4% and 6.0%, respectively (Fiori et al. 2012). Similarly, Pacific blue mackerel by‐products showed total omega‐3 fatty acids of 44.4%, with roe containing 11.3% EPA and 27.5% DHA, significantly higher than skin and heads (Ahmmed et al. 2021).

Fish processing industries generate solid fish waste that creates substantial environmental and economic challenges when improperly discarded. Uncontrolled disposal of fish waste into soil and aquatic environments increases biological oxygen demand (BOD), ammonia, sulfide, and methane emissions, accelerates eutrophication, produces offensive odors, and promotes pathogenic microbial growth, resulting in severe environmental degradation and public health risks (Kumar et al. 2025). Fisheries waste accumulation has also been associated with soil nutrient imbalance due to excessive nitrogen and phosphorus loading, which disrupts soil microbial ecology and contributes to groundwater contamination (Naseem et al. 2024). Economically, fisheries waste can lead to lost revenue in the seafood industry as valuable bioactive compounds such as collagen, gelatin, omega‐3 fatty acids, enzymes, peptides, and minerals are lost with the discarding of fish by‐products (Desalegn et al. 2026). Global food and seafood waste generation contributes substantially to greenhouse gas emissions, with food waste responsible for nearly 3.3 billion tonnes CO_2_‐equivalent emissions annually (FAO 2013). Consequently, fish waste valorization is very important to transform wastes into valuable products for a circular bioeconomy.

Extraction technology has a major impact on the efficiency and quality of recovered fish oil. Conventional wet rendering is the most common industrial method because of its simplicity and low operational cost; however, extended thermal exposure increases lipid oxidation, peroxide, and thiobarbituric acid reactive substances (TBARS) and reduces EPA/DHA stability (Shahidi and Ambigaipalan 2018). Solvent extraction offers higher lipid recovery and better omega‐3 yield, but solvent residues, environmental issues, and PUFA oxidation limit its food‐grade use (Swaidan et al. 2026). Enzymatic hydrolysis has been shown to be a potential alternative because proteolytic enzymes break down tissue matrices under mild conditions and release the oil while maintaining the integrity of omega‐3. The indices of oxidation were lower and EPA and DHA were better retained compared with thermal rendering. However, high enzyme cost, requirements for hydrolysis optimization, and emulsion formation to obtain hydrolyzed lipids remain challenging for food‐grade application (Annamalai and Sasikumar 2025).

Advanced green technologies, such as ultrasound‐assisted extraction (UAE), microwave‐assisted extraction (MAE), supercritical carbon dioxide (SC‐CO_2_), and pressurized liquid extraction, provide improved extraction efficiency and oil quality (Kalkan et al. 2025). UAE increases cavitation‐mediated cell disruption, shortens the extraction time, and reduces the solvent consumption while increasing the recovery of omega‐3. However, excessive ultrasonic intensity can lead to free radical formation and secondary lipid oxidation. MAE enhances heat transfer and extraction kinetics, but uncontrolled microwave exposure can result in damage to highly unsaturated fatty acids. SC‐CO_2_ extraction is one of the most efficient methods to obtain high‐purity omega‐3 oils, as it works under oxygen‐limited conditions and does not leave any toxic solvent residues, and it can also be used for selective concentration of EPA and DHA by pressure and temperature optimization (Xia et al. 2024; Desalegn et al. 2026). Despite these advantages, high capital investment and technical complexity limit widespread adoption and lower industrial scalability. Therefore, technology selection should balance oil quality, oxidative stability, environmental sustainability, scalability, and economic feasibility.

Extraction conditions substantially affect the physicochemical and functional properties of fish oil intended for encapsulation (Kumar and Chauhan 2025). Fish oils extracted under mild thermal and oxygen‐limited conditions exhibit lower peroxide values, anisidine values, and free fatty acid levels, leading to better encapsulation efficiency and storage stability, whereas oils subjected to excessive heat, light, or metal catalysts develop oxidation products that decrease bioactivity and affect sensory quality. Oxidized oils demonstrate lower encapsulation efficiency, weaker wall–core interactions, higher release kinetics, and lower antioxidant protection during storage (Strobel et al. 2020). The use of encapsulation technologies helps to enhance oxidative stability, mask undesirable sensory characteristics, enhance gastrointestinal delivery, and allows incorporation into dairy, bakery, meat, beverage, and nutraceutical systems (Advaitha et al. 2026). In general, extraction and encapsulation should be integrated as a continuous optimization strategy rather than treated as independent processing steps.

## 4 Stabilization and Encapsulation Techniques for Fish Oil

### 4.1 Nano Emulsion

**FIGURE 1:**
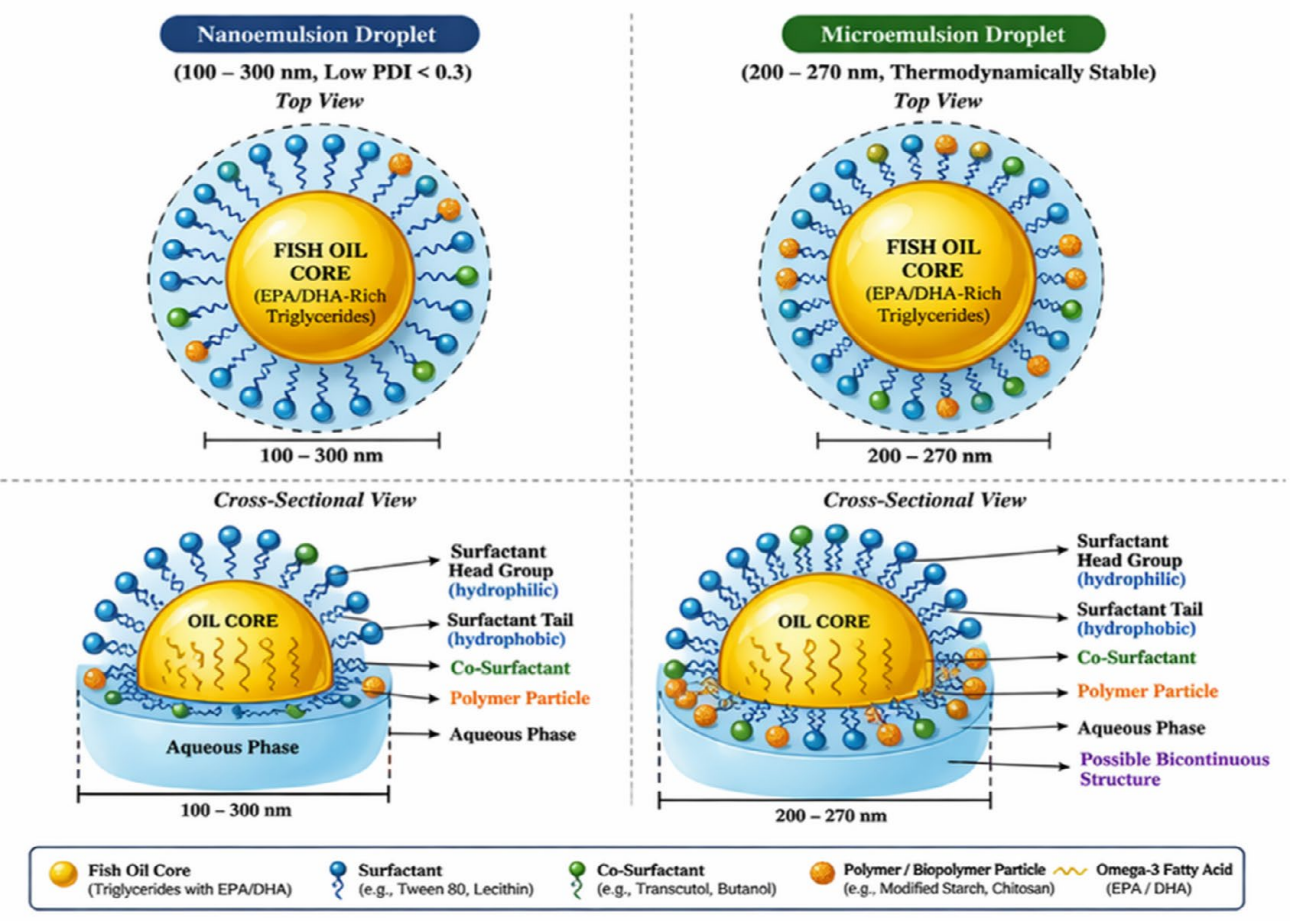
Nano/micro emulsion structures for fish oil encapsulation.

Nanoemulsion‐based encapsulation methods have emerged as one of the most promising fish oil stabilization methods for increased oxidative resistance, sensory acceptability, and incorporation of fish oil into aqueous‐based functional foods (Islam et al. 2023). Nanoemulsions can be formulated to have extremely small droplet sizes (usually < 200 nm) (Figure 1), which can increase interfacial surface area, water dispersibility, and often bioavailability of fatty acids such as omega‐3 EPA and DHA compared to bulk oil systems. They are particularly appropriate for fortified drinks, dairy products, and emulsified foods (Table 1) (Islam et al. 2023). More recent advances use high‐pressure microfluidization to produce Spirulina‐based nanoemulsions, which resulted in stable nanoscale droplets (92 nm) with a strong zeta potential and high resistance to oxidation upon long‐term storage, indicating both physical and chemical stability greater than that of controlled emulsions (Islam et al. 2023).

**TABLE 1:** Fish oil encapsulation methods.

Encapsulation types	Particle size	Encapsulation efficiency	Oxidative stability	Production cost	Industrial scalability	Sensory impact	Compatible food matrices	Main advantages	Main limitations	References
Nanoemulsions	< 200 nm	High (typically 80%–95%)	Moderate to high, depending on emulsifier and antioxidant system	Moderate	High; compatible with homogenization	Low impact due to small droplet size and improved dispersion	Beverages, dairy products, dressings, sauces	Excellent bioavailability, transparency, improved dispersion in aqueous foods	Potential oxidation due to large interfacial area; requires emulsifiers and energy‐intensive processing	(Kalladathvalappil Venugopalan et al. 2024)
Liposomes	50–200 nm	Moderate to high (60%–80%)	High because phospholipid bilayers protect omega‐3 lipids	High	Moderate; large‐scale production remains challenging	Minimal effect on flavor and texture	Dairy products, nutraceuticals, functional beverages	Good protection against oxidation and controlled release	High phospholipid cost, limited physical stability, scale‐up challenges	Amiri et al. (2023), Ahmad et al. (2025)
Micelles	150–200 nm	Moderate	Moderate	Low to moderate	High	Very low sensory impact due to nanoscale size	Clear beverages, liquid supplements	Excellent solubilization of hydrophobic omega‐3 oils and enhanced absorption	Limited loading capacity and reduced long‐term stability	Zhou et al. (2025)
Nano/Microgels	100–500 nm	High	High due to dense polymeric network and physical barrier properties	Moderate	Moderate	Generally low impact; can improve texture in some products	Yogurt, dairy gels, bakery products, semi‐solid foods	Strong protection against oxidation and controlled release during digestion	More complex formulation and processing requirements	Li and Liu (2024)
Electrospun/Electrosprayed fibers	10 nm–few mM	Very high (> 80%–90% in optimized systems)	Very high because omega‐3 oils are entrapped in dry polymer matrices	High	Currently limited; mostly pilot‐scale applications	Minimal flavor impact due to efficient encapsulation	Powders, nutraceuticals, dry food systems	Excellent protection of oxidation‐sensitive lipids and solvent‐free/low‐temperature processing	Limited industrial adoption and relatively high production costs	Rahmani‐Manglano et al. (2024), García‐Moreno et al. (2016)

Another recent study by Shanuke et al. (2025) produced fish oil nanoemulsions incorporated into yogurt, in which encapsulation decreased the total oxidation (TOTOX) values and maintained high EPA and DHA levels for 21 days of storage, which implies that nanoemulsions are practical for real food matrices and may improve the sensory quality as well as the nutritional delivery. Nanoemulsions of tuna fish oil (TFO) co‐encapsulating curcumin and resveratrol as natural antioxidants were prepared with modified starch carriers. The sample demonstrates that optimized formulations had a smaller droplet size and greater stability to oxidation at different storage temperatures, which suggests that the strategic co‐loading of antioxidants can substantially delay lipid peroxidation in nanoemulsions, which is vital for functional food enrichment (Shehzad et al. 2021).

Multi‐modified β‐lactoglobulin fibril–stabilized emulsions have a quantitatively superior performance over conventional protein or surfactant‐stabilized systems, with encapsulation efficiencies exceeding 90% and peroxide value reductions typically greater than 40%–60% under accelerated storage conditions compared to native β‐lactoglobulin interfaces (Zhang et al. 2025). Importantly, interfacial modification led to improved digestive functionality, as evidenced by a 1.5–2.0‐fold increase in post‐digestion micellar incorporation of EPA/DHA and a corresponding increase in Caco‐2 cellular uptake exceeding 30%–50%, suggesting an improvement in trans‐epithelial transport efficiency. These results are in line with other recent encapsulation systems, such as nanoemulsions and hollow solid lipid nanoparticles, which report encapsulation efficiencies in the range of 80%–90% and intestinal release levels of 60%–70% (Paul et al. 2024).

However, current research on fish oil nanoemulsions is still limited by insufficient in vivo and simulated gastrointestinal validation that will clarify the impact of encapsulation on post‐digestion bioavailability and functional efficacy. Limited studies offer integrated, quantitative assessments of sensory quality, nutritional value, and health‐related functionality under realistic storage and processing conditions. Future work should employ standardized, comparative designs that evaluate emulsifier type, antioxidant co‐loading, and droplet size to clarify the structure–function relationships that govern stability and bioactivity. To advance toward commercial application, nanoemulsion systems will need to be coupled with controlled‐release strategies, and the bioavailability, functional performance, and consumer acceptability will need to be rigorously validated.

### 4.2 Liposomes

**FIGURE 2:**
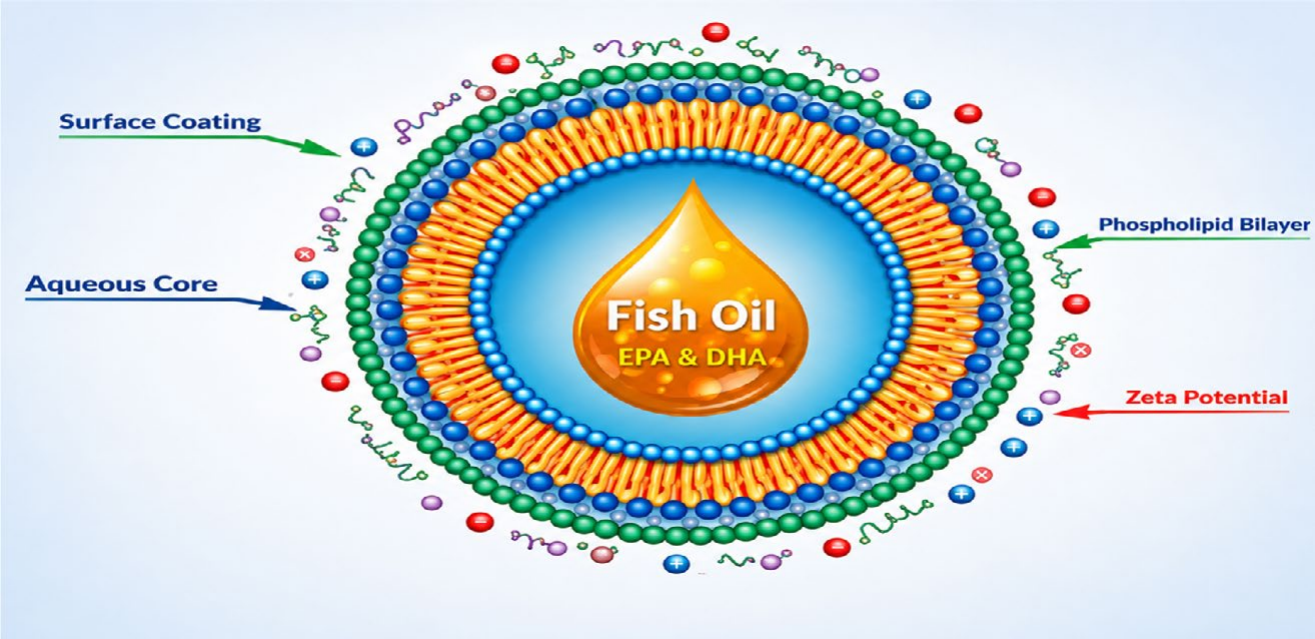
Core structures of liposome encapsulation of fish oil.

Omega‐3 polyunsaturated fatty acids (PUFAs), including eicosapentaenoic acid (EPA) and docosahexaenoic acid (DHA) found in fish oil, have limited applications in foods and biomedical products due to their poor water solubility and susceptibility to oxidation. Liposomes are one of the encapsulation methods to stabilize PUFAs against oxidation, typically comprised of phospholipids organized into bilayer structures that are organized into concentric rings (Figure 2). They contain both non‐polar and polar regions that can be used to encapsulate hydrophilic and hydrophobic substances. The liposomes, or phospholipid bilayer vesicles, were used as carriers to stabilize and increase the bioavailability of fish oil (Ahmad et al. 2025). Liposomal encapsulation can increase oxidative stability and protect PUFAs during storage, but it has limitations in large‐scale production and physical stability. The particle sizes range widely from 50 to 200 nm with improved oxidative resistance in smaller liposomes, which are thought to result from both size and surface charge effects (Table 1) (Venugopalan et al. 2021).

A novel liposome‐like self‐micro emulsifying drug delivery system (SMEDS) for fish oil EPA and DHA with improved bioavailability was developed using a Box–Behnken statistical design to optimize the lecithin, PEG, propylene glycol ester, and polysorbate ratios. The sample exhibited a particle size of 100–300 nm, low polydispersity (PDI 0.149–0.303), and stable zeta potential (−32 to −28 mV), which are characteristics of a narrow size distribution and colloidal stability. Pharmacokinetic evaluation in Wistar rats demonstrated a 13.2‐fold increase in EPA and 4.7‐fold increase in DHA bioavailability compared to conventional fish oil. This study had a controlled design and in vivo assessment, but a weakness is the lack of human clinical data and potential translation issues, as rodent metabolism does not always reflect human absorption (Ahmad et al. 2025).

Amiri et al. (2023) also demonstrated that liposomal encapsulation can enhance the stability of marine bioactives, but the performance of liposomes was highly dependent on the coating methods. Uncoated liposomes rapidly destabilized with considerable particle aggregation and leakage during storage, whereas chitosan and whey‐protein coated systems maintained smaller particle sizes, improved zeta potential, and higher structural integrity. This suggests better colloidal stability; oxidative markers (peroxide value and TBARS) were consistently lower in coated systems, confirming a measurable reduction in lipid oxidation, although the improvement was moderate, as oxidation still progressed over time, and encapsulation efficiency was also higher in multilayer systems, approaching values of 90%–97% in similar formulations, which suggests improved retention of EPA/DHA. However, the study lacks kinetic modeling of oxidation, long‐term storage validation, and bioaccessibility assessment. Additionally, the increased structural complexity of multilayer coatings introduces formulation variability without clear optimization criteria and may compromise release behavior, limiting the predictability and scalability of the system for functional food applications.

Overall, liposomal and related nanocarriers offer better oxidative protection of omega‐3 fish oil compared with free oil, as indicated by slower peroxide value increases, higher retention of EPA/DHA, better sensory outcomes in food matrices, high encapsulation efficiencies (often > 90%), and enhanced storage stability (Table 1). It will be important for future work to prioritize standardized quantitative reporting of encapsulation metrics, comparative studies against alternative delivery systems, and human bioavailability studies to validate animal model findings. Furthermore, new technologies, such as bio‐coatings designed for targeted release in the GI tract and stimuli‐responsive liposomes, may overcome some of the current challenges with targeted delivery, and rigorous kinetic modeling and in vitro digestion will be needed to successfully integrate liposomal fish oil formulations into diverse food matrices.

### 4.3 Micelle

**FIGURE 3:**
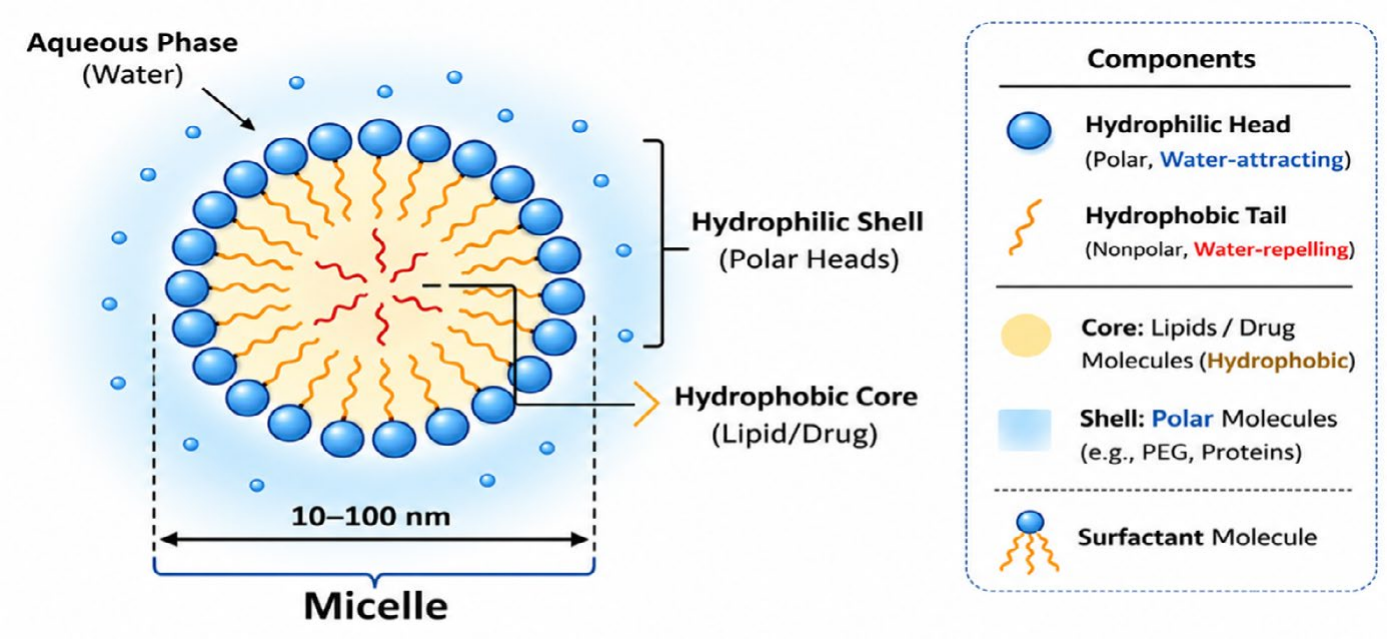
Structure of micelle encapsulation of fish oil.

The protection of fish oil from oxidation and improved bioaccessibility of omega‐3 PUFAs are attracting much interest to micellar encapsulation and related colloidal delivery systems. Micelles are self‐assembled structures of amphiphilic molecules or amphiphilic polymers in aqueous media with hydrophobic cores that solubilize lipophilic substances in water, regulated by critical micelle concentration (CMC) and surfactant composition, which can incorporate fish oil triglycerides in the hydrophobic core and provide colloidal stability due to a hydrophilic shell (Figure 3). Nevertheless, the application of micelles to fish oil encapsulation is limited compared with other nano‐ and microencapsulation strategies due to limited loading capacity and minimum long‐term stability (Perumal et al. 2022).

A recent randomized crossover human pharmacokinetics study evaluated micellar formulations of fish oil compared with standard and enteric‐coated fish oil capsules. In this work, the micellar fish oil formulation achieved significantly higher blood concentrations and area under the curve (AUC) for EPA and total n‐3 fatty acids approximately five to seven times greater EPA AUC than other formulations at higher nominal doses and incremental blood levels of total n‐3 FAs. These results quantitatively suggest that micelles or self‐emulsifying systems markedly enhance absorption through improved solubilization and intestinal uptake mechanisms compared with conventional delivery systems (Ibi et al. 2024).

### 4.4 Nano−/Micro‐Gels

**FIGURE 4:**
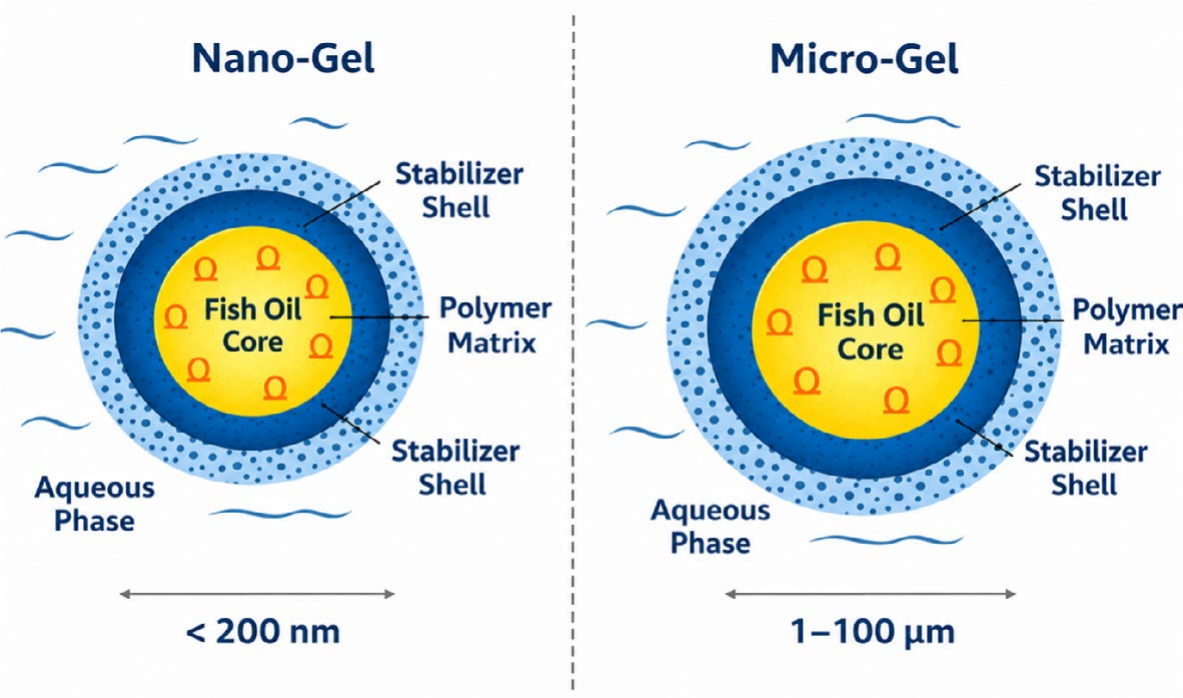
Nano/micro gel encapsulation structures of fish oil.

Encapsulation in biopolymer gels such as gelatin or polysaccharide networks has been explored to protect omega‐3 polyunsaturated fatty acids against oxidation while improving dispersibility in aqueous environments. Nano/Microgels consist of small spherical particles that have a network of cross‐linked biopolymers inside, where an aqueous solution fills the pores (Figure 4) (Desalegn et al. 2026). Gelatin, derived from animal collagen, forms a three‐dimensional network capable of entrapping lipophilic fish oil droplets and thereby reducing oxidative degradation and masking undesirable odors that typically limit fish oil use in food formulations. Various gelatin types (e.g., porcine, bovine, and fish gelatin) differ in gel strength, melting behavior, and film‐forming capacity, which modulate encapsulation efficiency and product stability. Fish gelatin often exhibits favorable biocompatibility and consumer acceptance but presents weaker mechanical properties that can compromise protective efficacy if not cross‐linked or blended with other polymers. Gel matrices influence stability attributes of encapsulated fish oil, emphasizing oxidative stability and functional quality retention (Mude 2024).

A recent study on fish‐oil hydrogel encapsulation has demonstrated that cold‐set fish gelatin encapsulation with ionically cross‐linked alginate has been shown to improve both structural integrity and oxidative protection compared to mono‐component gels. In dual hydrogels, cations (Fe^3+^ and Ba^2+^) affected textural properties (hardness, 28.6 ± 0.4 N, and gumminess, 6.9 ± 0.3 N, respectively) and water activity (0.94) restricted moisture‐driven degradation. Free fatty acid release in simulated intestinal conditions suggests that ionic composition modulates release profiles, with non‐Na^+^ ions encouraging higher FFA release (Chen et al. 2024). Other encapsulation matrices, such as electrospun pullulan/sodium caseinate nanofibers or genipin‐crosslinked gelatin‐polysaccharide coacervates, show peroxide values ranging from 2.4 to −4.8 mM under refrigerated or accelerated conditions and FFA release from 48.9% ± 1.5% to 64.8% ± 2.9%, which demonstrates a significant effect on both oxidative stability and bioaccessibility (Dabora et al. 2025). Overall, dual gelatin‐alginate hydrogels offer significant enhancements in structural and oxidative performance, but standardized long‐term oxidation kinetics and comparative quantitative metrics across encapsulation systems are lacking, which makes rigorous benchmarking difficult.

The NE ω3 gel was characterized by dynamic light scattering analysis showing a homogeneous nanoemulsion with an average particle size of 287 nm and a narrow size distribution (PDI 0.29), and the thermoreversible gel exhibited a controlled release of fish oil with > 96% release after 10 h, which supports controlled release behavior. In vivo rat models exhibited up to 21% reduction in capsular thickness and significant decreases in fibrosis and myofibroblast counts compared to controls, suggesting therapeutic benefits to reduced inflammation for postoperative applications. However, it lacks biomedical relevance for food applications, and the rapid release of 10 h is not suitable for sustained absorption (Paul et al. 2024).

Gel encapsulation offers biocompatible matrices that improve the stability of fish oil, mask off flavors, and allow controlled release in foods and nutraceuticals (Table 1). Micro/nano gel encapsulation could behave a strong protection against oxidation and controlled release during digestion; however, complex formulation and processing requirements exist. Hybrid nanofiber gels offer high surface area and good lipid‐matrix compatibility for protection and possibly bioactivity. Inconsistencies in oxidative stability metrics, lack of in vivo digestibility data, mechanical weaknesses in natural gels, and overemphasizing in vitro stability with minimal evidence of bioavailability or physiological uptake limit comparability and industrial application. Standardization of oxidative and digestion assays, assessments of bioavailability, development of multifunctional or stimuli‐responsive gels, and clarification of performance by linking gel structure, rheology, and release kinetics, as well as translational studies addressing industrial scalability, sensory quality, and consumer acceptance.

### 4.5 Electrospinning

The highly unsaturated lipid fish oil is a potential candidate for food fortification, and its oxidative stability and functional performance can be enhanced by encapsulation using electrospinning. Electrospinning is a nanofabrication technique that can produce continuous polymeric fibers in the range of tens of nanometers to a few micrometers (Table 1). During application to fish oil, a lipid containing polyunsaturated omega 3 fatty acids, such as EPA and DHA, electrospinning generates a fibrous network with a high surface area that can physically block access to oxygen and environmental stressors, reducing lipid oxidation and enhancing functional stability. Encapsulation efficiency, fiber morphology, and oxidative protection are strongly dependent on the choice of polymer matrix, processing conditions, and oil loading. Electrospun fibers also enable controlled release and potential modulation of bioactive interactions that are beneficial for food and nutraceutical delivery systems (Yang et al. 2017).

Coaxial electrospinning can also be used to produce core‐shell structures, where the fish oil is encapsulated in an inner core, and the shell is a polymer that acts as a barrier to oxygen diffusion to the core, slowing peroxidation reactions. This greatly enhances oxidative stability of the oil when compared to single/monoaxial fibers for example, 96.9% encapsulation efficiency and lower oxidation relative to single fibers in a comparable system (Yang et al. 2017). Core‐shell nanofibers have also been investigated using edible polymer systems, such as fish oil–gelatin bilayer membranes that successfully incorporate both hydrophobic (fish oil) and hydrophilic (vitamin C) components.

In another comparison of fish‐oil encapsulation, monoaxially electrosprayed systems produced using emulsion electrospinning with carbohydrate matrices achieved encapsulation efficiencies of 69%–72% compared to 84%–90% for spray‐drying and 53%–59% for coaxial electrospraying (Rahmani‐Manglano et al. 2024), which shows that monoaxial systems can moderately entrap oil but with less retention than conventional methods under the same formulations. However, monoaxially electrosprayed capsules had higher initial oxidation levels immediately after production, characterized by elevated secondary volatile oxidation products, likely because of prolonged processing time and exposure during emulsification (Rahmani‐Manglano et al. 2024; García‐Moreno et al. 2016).

Furthermore, another work on electrospun zein fibers has shown high encapsulation efficiencies up to 91%–96% at 30% oil loading, but the oxidative stability gains were observed only relative to free oil, not directly compared with core‐shell structures, and increasing oil content correlated with larger fiber diameters and potential surface exposure of lipids. Monoaxial systems reveal that physicochemical parameters such as polymer type and emulsion stability strongly influence fiber morphology and protective performance, and that emulsifier choice can impact droplet size and fiber quality. While monoaxial fibers are easier to fabricate, oxidation suppression is often less pronounced unless additional stabilizing agents (e.g., antioxidants) are incorporated (Liu et al. 2021).

Hybrid nanofibers, which blend multiple polymers (e.g., pullulan with sodium caseinate) and may include antioxidants, have demonstrated improved fish oil protection compared with simpler monoaxial fibers. One recent study, Dabora et al. (2025) work on encapsulating fish oil in pullulan/sodium caseinate nanofibers provides a valuable quantitative and mechanistic evaluation. In this study, electrospinning was used to fabricate nanofibers containing 0%, 5%, or 10% fish oil, and their physicochemical and functional properties were compared. Increasing fish oil content increased fiber diameter and surface hydrophobicity, and at 5% oil the system achieved optimal antioxidant activity (measured by DPPH, ABTS, and total phenolic assays) and enhanced oxidative stability, reducing hydroperoxide formation from 8.6 to 4.8 mM over 15 days at 60°C. The study showed that the 5% formulation balanced encapsulation efficiency (~84%) and functional performance, while the 10% formulation exhibited structural heterogeneity and lower bioactivity, likely due to polymer saturation and non‐uniform oil distribution. However, the study lacked a non‐electrospun control using the same biopolymer matrix, which limits isolation of electrospinning's specific structural advantages, and did not assess long‐term storage or sensory attributes relevant to food applications.

The importance of electrospinning for fish oil encapsulation lies in its ability to produce nanofibrous carriers with high surface area, tunable porosity, and the potential to incorporate multiple functional agents, creating diffusion barriers that slow lipid peroxidation and preserve the nutritional value of omega‐3 oils (Rahmani‐Manglano et al. 2024). Incorporation of antioxidants or co‐functional components into the fiber matrix can further enhance oxidative stability and enable controlled release. However, limitations remain, including a lack of direct comparisons with conventional encapsulation methods, insufficient long‐term storage and sensory data, challenges in polymer selection, oil loading, and fiber defects, as well as potential oxidative stress during the electrospinning process itself. Future research should address these gaps by systematically comparing electrospinning with other techniques using standardized metrics, optimizing hybrid polymer and antioxidant formulations, developing scalable electrospinning technologies, and evaluating in vivo bioavailability, sensory impact, and interactions with complex food matrices to fully realize the potential of electrospun fish oil for functional food applications.

## 5 Controlled Release and Bioavailability of Encapsulated Fish Oil

Fish oil encapsulation systems have been shown to have markedly different release profiles in simulated gastrointestinal environments depending on the composition of the matrix, the structural architecture, and the interactions with digestive enzymes. Jiang et al. (2025) showed that encapsulated DHA had a much lower free fatty acid (FFA) release (12.28 ± 0.12 mmol g^−1^ fish oil) than bulk oil (16.24 ± 1.35 mmol g^−1^) and emulsified formulations (18.18 ± 1.07 mmol g^−1^) during in vitro digestion (Table 2). This demonstrated that encapsulated fish oil delivery systems can delay initial lipid hydrolysis and provide a more controlled release profile under simulated gastrointestinal conditions. The associated apparent permeability coefficient (*P*_app_) for DHA (1.69 ± 0.06 × 10^−5^ cm s^−1^) was also between bulk and emulsified systems, and ex vivo rat intestinal absorption was 10.7% for DHA compared to 16.2% for bulk oil and 17.3% for emulsified systems.

**TABLE 2:** The controlled releases of encapsulated fish oil in the digestion system.

No.	Encapsulation system	Encapsulation efficiency (%)	Gastric % release	Intestinal % release	Release mechanism/Model	References
1	Gelatin‐glycerin encapsulated DHA vs. bulk & emulsified	Not reported	Low release in gastric phase	Higher intestinal release, Encapsulated: 12.28 ± 0.12; Emulsified: 18.18 ± 1.07, Papp (dialysis): 1.69 ± 0.06 × 10^−5^ cm s^−1^ (encapsulated)	Diffusion‐mediated release; dialysis & ex vivo permeability	Jiang et al. (2025)
2	BBG‐genipin‐AP coacervate powders	90.9 ± 0.7 (highest)	Transforms to emulsion in gastric phase (minimal measured)	GA: 64.8 ± 2.9; SA: 55.0 ± 3.1; CMC: 48.9 ± 1.5 (2 h gastric +2 h intestinal)	pH‐triggered matrix swelling and enzymatic erosion	Peng et al. (2024)
3	Microfluidic ternary microcapsules (SA‐GA‐LM)	Not reported	< 7% release in SGF within 120 min	77% release in SIF within 120 min	pH‐responsive ionic gel matrix	Xie et al. (2025)
4	Protein‐based cryogel oleogel matrix	Not explicitly stated	Lower gastric FFA release than control	62.17 ± 2.76 (cryogel) vs. 83.61 ± 1.42 (WPI)	Dense protein network limits enzymatic access	Purba et al. (2025)
5	Phospholipid‐assisted microcapsules	90% EE	Minimal early gastric release	Progressive FFA release (not numerically specified in abstract)	Phospholipid interface facilitating controlled hydrolysis	Guo et al. (2025)
6	Rice bran protein fibrils + xanthan gum microcapsules	85%–92%	Significantly reduced release in gastric conditions	Controlled intestinal release higher than gastric	Dense fibrillar/polysaccharide barrier limiting diffusion	Tang et al. (2022)
7	Solid lipid particle encapsulation (wax)	92.5% with carnauba wax	Not reported	Enhanced bioaccessibility vs. non‐encapsulated (6.1%–11.2%)	Hydrophobic solid lipid matrix restricting early release	Okur et al. 2025

Mechanistically, controlled release in such systems occurs as a result of physicochemical barriers to lipase access and water penetration; protein‐based matrices slow the diffusion of triglycerides to digestive enzymes and reduce the interfacial surface area available for hydrolysis. Peng et al. (2024) found that crosslinked matrices have lower water activity (0.45 ± 0.01) (Table 2), altered density and packing parameters, and reduced oxidation indicators that correspond to reduced lipid mobility and delayed enzymatic degradation; higher tapped densities and compaction likely limit matrix swelling and enzyme permeation, resulting in more sustained release relative to non crosslinked powders.

Xie et al. (2025) designed plug‐and‐play microfluidic chips to produce monodisperse fish oil microcapsules with tunable shell thickness (163–592 μm) and high structural homogeneity. The ternary wall matrix of sodium alginate, gum arabic, and low‐methoxyl pectin formed ionically crosslinked networks that provided oxidative protection and pH‐responsive permeability. In vitro digestion assays revealed low gastric leakage (< 7% release in simulated gastric fluid) and rapid intestinal delivery (approximately 77% cumulative release after 120 min in simulated intestinal fluid), suggesting effective site‐specific release controlled by pH‐induced polymer relaxation and erosion. However, combining ionic gelation with microfluidic control offers a quantitative approach to encapsulate design and controlled targeted release of omega‐3 with enhanced physicochemical stability.

Chen et al. (2025) created fish oil microcapsule powders by fish gelatin–alginate complex coacervation followed by intra‐particulate genipin crosslinking to control oxidative stability and intestinal release behavior. The genipin‐induced covalent network reinforced the gelatin–alginate matrix, inhibiting premature lipid leakage under gastric conditions but allowing progressive matrix swelling and enzymatic degradation during simulated intestinal digestion, resulting in a logarithmic increase in free fatty acid release confirmed by in vitro digestion, suggesting a diffusion‐ and erosion‐controlled release mechanism controlled by crosslinked polymer relaxation. Rice bran protein fibrils in combination with xanthan gum form a dense interfacial network around fish oil droplets, providing a diffusion‐mediated barrier to limit rapid lipase access during the gastric phase and slow release (Tang et al. 2022).

Protein‐based cryogel–oleogel hybrids with fish gelatin and whey protein isolate were demonstrated to reduce cumulative intestinal FFA release from 83.6% ± 1.4% for WPI‐only to 62.2% ± 2.8% (Table 2) (Purba et al. 2025), which demonstrates that dense, crosslinked networks restrict enzyme access and slow lipid hydrolysis, while maintaining gastric stability. In a similar manner, supercritical CO_2_ encapsulated fish oil in wax‐based particles increased EPA/DHA bioaccessibility from 6.1% (candelilla wax) to 11.2% (carnauba wax) (Okur et al. 2025), which demonstrates that solid lipid matrices resist premature oxidation and promote slow, digestion responsive release. Collectively, these methods demonstrate that matrix composition and structural integrity are critical for controlled, intestinal‐targeted omega 3 delivery.

The encapsulated fish oil methods can limit lipid hydrolysis in the gastric phase, with release increasing under intestinal pH, bile salt action, and enzymatic activity. The extent of controlled release depends on wall matrix composition, crosslinking degree, and structural morphology, while coacervates, oleogels, and solid lipid particles exhibit variable but mechanistically consistent patterns of delayed digestion, moderated lipase access, and altered absorption kinetics. In terms of mechanisms, delayed release is controlled by diffusion limitations, matrix swelling and erosion kinetics, and biochemical responsiveness to gastrointestinal conditions, making encapsulation strategies key modulators of in vivo bioavailability and targeted nutrient delivery.

## 6 Antioxidant Mechanisms of Omega‐3 Bioactive Lipids in Functional Foods

### 6.1 Antioxidant Mechanisms of Omega‐3 Bioactive Lipids

**FIGURE 5:**
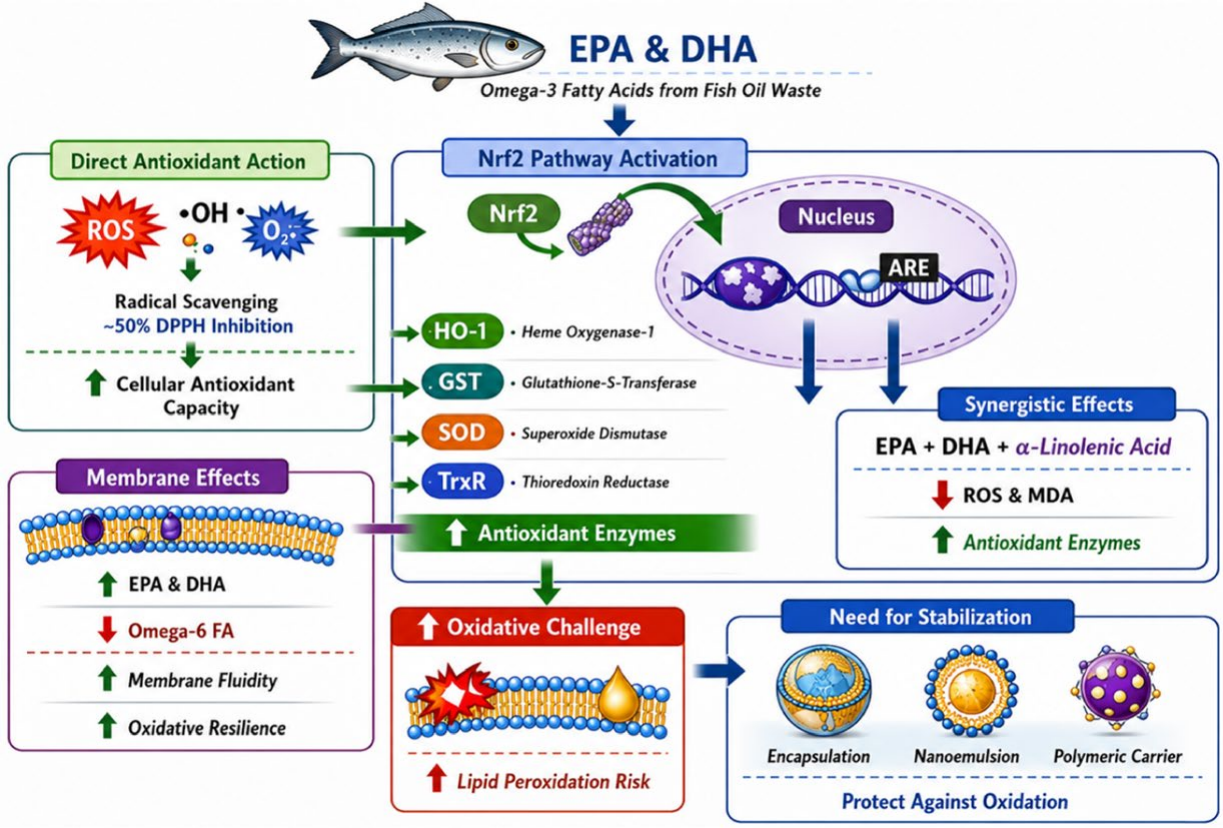
Antioxidant molecular mechanism of action of omega‐3 fatty acids.

Omega‐3 bioactive lipids in fish waste, especially docosahexaenoic acid and eicosapentaenoic acid, are natural antioxidants. Their functional efficacy as natural food antioxidants in fish‐oil‐fortified foods is attributed to their ability to scavenge free radicals, inhibit reactive oxygen species (ROS) formation, and modulate cellular antioxidant enzymes through activation of redox‐sensitive transcription pathways. This functionality contributes to their antioxidant functionality in biological systems and oxidative stability in food matrices. Molecularly, omega‐3 fatty acids activate the Nrf2 antioxidant signaling pathway that controls the expression of endogenous antioxidant enzymes, including glutathione‐S‐transferase, catalase, and superoxide dismutase. EPA and DHA have been shown to decrease intracellular ROS and to upregulate Nrf2‐dependent genes, such as heme oxygenase‐1 and thioredoxin reductase, and silencing the Nrf2 gene eliminates these protective effects, suggesting that the antioxidant activity of omega‐3 lipids is mainly mediated through Nrf2 signaling (Figure 5) (Sakai et al. 2017).

Moreover, omega‐3 fatty acids can also directly scavenge free radicals, and EPA and DHA have been shown to have moderate direct antioxidant capacity compared to classical antioxidants in cell‐based antioxidant assays (49.7% and 50.5% DPPH inhibition, respectively) (Aldhafiri 2022). These effects are complemented by increased intracellular glutathione levels and induction of antioxidant response element (ARE)‐regulated genes, which collectively enhance cellular antioxidant defense systems (Saw et al. 2013). Omega‐3 lipids also modulate oxidative stress in addition to enzymatic regulation; dietary supplementation with fish oil increases the incorporation of DHA and EPA into membranes, reducing the proportion of pro‐oxidant omega‐6 fatty acids and altering membrane fluidity and oxidative resilience (Miralles‐Pérez et al. 2021). Combinations of omega‐3 fatty acids have been shown to increase antioxidant capacity in recent cellular studies, with combinations of 145 μM EPA, 405 μM α‐linolenic acid, and 551 μM DHA having the greatest effect in increasing antioxidant enzymes and reducing malondialdehyde and intracellular ROS in hepatocytes (Wang et al. 2024).

Overall, the antioxidant mechanisms of omega‐3 bioactive lipids involve a combination of direct radical scavenging, activation of cellular antioxidant pathways, modulation of membrane lipid composition, and synergistic interactions among fatty acids. However, the susceptibility of these lipids to oxidative degradation necessitates stabilization strategies, particularly when derived from fish‐processing waste and incorporated into functional foods.

### 6.2 Antioxidant Functionality of Fish Oil‐Fortified Foods

Fortification of foods with marine fish oils rich in long‐chain omega‐3 polyunsaturated fatty acids (PUFAs) such as eicosapentaenoic acid (EPA) and docosahexaenoic acid (DHA) has emerged as an established method to improve the nutritional quality of foods and to deliver health promoting lipids using foods. However, the highly unsaturated nature of long chain PUFAs makes them intrinsically vulnerable to oxidation, a process that compromises food safety and organoleptic quality, and reduces the efficacy of their bioactive properties. Lipid oxidation, initiated by exposure to oxygen, light, heat, or pro‐oxidative metals, rapidly generates primary (hydroperoxides) and secondary (aldehydes, ketones) oxidation products that compromise both safety and organoleptic properties of fortified matrices. This instability is quantifiable by standard measures such as peroxide value (PV), thiobarbituric acid‐reactive substances (TBARS), and TOTOX indices (combined primary and secondary oxidation) that provide objective oxidation profiles during processing and storage (Dragoev 2024).

The oxidation kinetics of free and structurally protected fish oil have been well documented, and it was found that encapsulation of fish oil with protein–polysaccharide systems markedly changed its oxidative stability under accelerated conditions. The peroxide value (PV) remained at 4.8 ± 0.23 meq/kg after 4 weeks of storage for encapsulated PUFA, while unencapsulated PUFA reached 43.8 ± 0.11 meq/kg. This evidenced a clearly slower oxidation rate, and induction point (IP) measurements via Rancimat, a kinetic index of oxidation resistance, showed a nearly ten‐fold increase (5.12 ± 0.13 h for encapsulated PUFA and 0.54 ± 0.12 h for unencapsulated oil) under accelerated conditions, confirming the resistance of encapsulated oil to oxidative initiation (Kalladathvalappil Venugopalan et al. 2024).

Fish oil fortification in the form of nanoemulsions within yogurt matrices resulted in improved omega‐3 retention (EPA + DHA post‐storage retention up to 71.75% ± 2.84%) and lower total oxidation values (TOTOX). This could indicate that emulsification strategies reduce oxidative stress markers more effectively than bulk oil addition and enable higher levels of functional lipid incorporation without compromising on product quality metrics (Shanuke et al. 2025). The associations between food matrix, antioxidant intervention, and functional oxidative outcomes demonstrate a multidimensional paradigm in fish oil fortification where encapsulation and antioxidant addition can markedly reduce oxidation while retaining health‐beneficial lipids; however, optimization must reconcile sensory acceptability, processing compatibility, and nutritional objectives. The studies consistently show that formulation can deliver clinically relevant doses of EPA and DHA.

The antioxidant functionality in fortified foods originates from both physical barriers to oxygen and radical diffusion. The chemical scavenging of peroxyl and alkoxyl radicals by phenolic and tocopherol‐type compounds, which inhibit propagation of lipid peroxidation and maintain functional integrity of omega 3 PUFAs during processing and storage, is important. Thus, the antioxidant functionality of fish oil fortified foods is based on intricate interactions between lipid chemistry, food matrix, antioxidant systems, and processing conditions. Recent investigations indicate that encapsulation can improve oxidative stability, that combinations of natural antioxidants can offer synergistic protection, and that fortified products can supply high concentrations of omega 3s with minimal oxidative degradation when formulated appropriately.

## 7 Antimicrobial Mechanisms of Omega‐3 Bioactive Lipids in Functional Foods

### 7.1 Antimicrobial Mechanisms of Omega‐3 Bioactive Lipids

Omega‐3 bioactive lipids such as EPA and DHA have demonstrated antimicrobial activity at the molecular and cellular levels, which greatly enhances their potential utility in fortified functional foods. The antimicrobial effects of these long‐chain polyunsaturated fatty acids are due to their interactions with microbial cell structures and biofilm architecture rather than specific enzyme inhibition, and several peer‐reviewed studies have demonstrated that EPA and DHA reduce microbial viability and biofilm formation in both Gram‐positive and Gram‐negative bacteria. In vitro multispecies biofilm models have demonstrated that EPA and DHA at concentrations of 100 μM cause significant reductions in biomass and live bacterial counts, demonstrating broad‐spectrum inhibitory potential against pathogens such as *Streptococcus oralis* , *Fusobacterium nucleatum* , and *Porphyromonas gingivalis* in subgingival biofilms. Evidence of direct membrane‐targeted effects of EPA and DHA, which can lead to bacterial membrane damage (Ribeiro‐Vidal et al. 2020).

**FIGURE 6:**
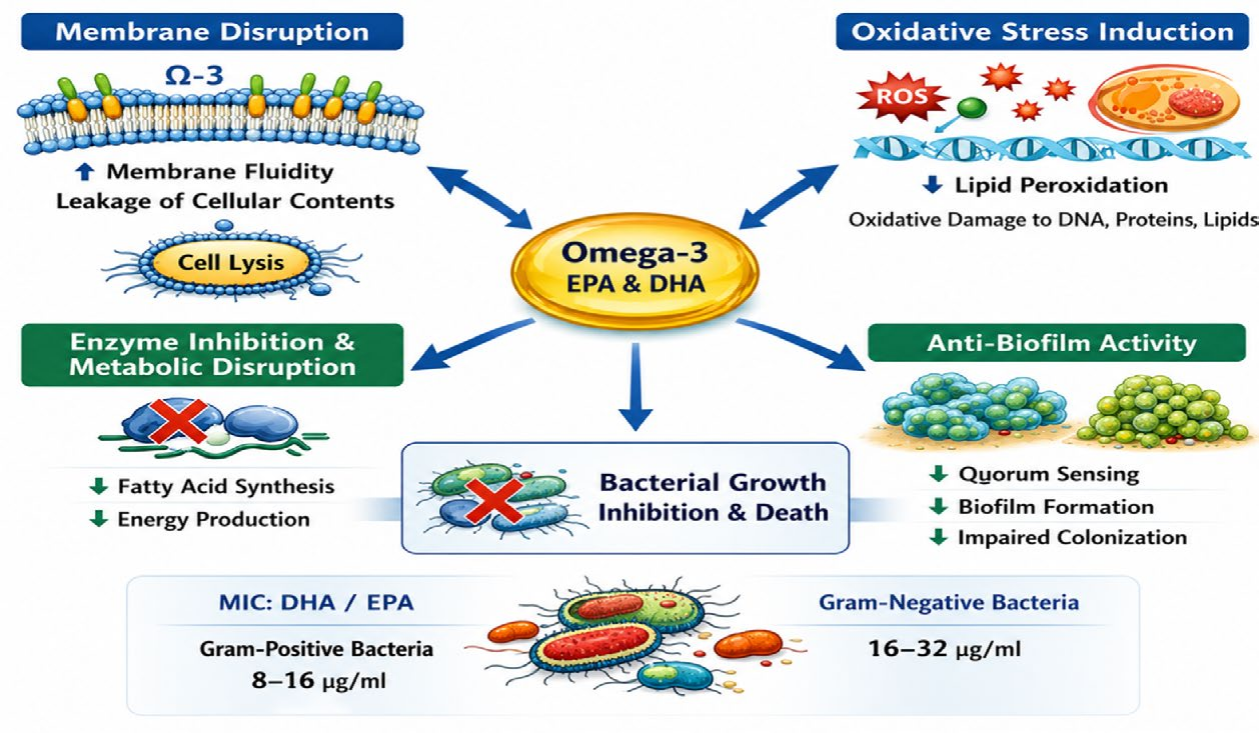
Antimicrobial mechanism of action of omega‐3 fatty acids.

Mechanistically, the insertion of unsaturated omega‐3 fatty acids into microbial membranes is thought to disrupt lipid packing and increase permeability, resulting in the detergent‐like insertion, causing leakage of essential intracellular contents and disruption of energy‐conserving processes such as electron transport and oxidative phosphorylation, resulting in loss of viability (Figure 6) (Obukhova and Murzina 2024). In addition to their effects on planktonic bacteria, EPA and DHA also significantly inhibit biofilm formation and bacterial virulence factor expression, such as in studies with *Staphylococcus aureus* , in which exposure to omega‐3 lipids at subinhibitory concentrations inhibited biofilm formation and reduced the expression of virulence genes like hla, which reduces hemolytic activity and increases susceptibility to immune clearance (Kim et al. 2018). In controlled experimental systems, EPA and DHA exhibit minimum inhibitory or biofilm reduction effects in the low micromolar range. EPA was slightly more antimicrobial than DHA against clinical bacterial isolates with complete killing or significant reduction observed at higher test concentrations (Coraça‐Huber et al. 2021; Tables 1 and 2).

Encapsulation technologies have demonstrated substantial potential for improving the antimicrobial efficacy, stability, and bioavailability of lipid‐based bioactive compounds. The antimicrobial mechanisms of encapsulated fish oil and lipid‐based nanoformulations involve multiple biochemical pathways. Omega‐3 fatty acids such as DHA and EPA can integrate into microbial phospholipid bilayers, altering membrane fluidity, permeability, and structural integrity. This disruption causes leakage of ions, proteins, and nucleic acids, ultimately impairing microbial metabolism and replication (Alavi et al. 2022). Khoshnood et al. (2023) reported that amoxicillin‐docosahexaenoic acid (DHA) encapsulated chitosan‐alginate nanoparticles formed in an ionic gelation method exhibited markedly enhanced biocidal activity against *Helicobacter pylori* . The nanoparticles showed stronger antibacterial performance than free amoxicillin due to synergistic effects between DHA and the antibiotic. DHA disrupts bacterial membrane integrity by increasing membrane fluidity and oxidative stress. Encapsulation within chitosan‐alginate matrices improved stability in gastric conditions and enabled controlled release at the infection site.

According to Azmi et al. (2022) study demonstrated that the nanoemulsion of fish by‐product oil and lemon oil (NE‐FLO) possessed broad‐spectrum antimicrobial activity against both Gram‐positive and Gram‐negative bacteria. The nanoemulsion effectively inhibited all fourteen tested bacterial strains, producing inhibition zones of 2–8.67 mm for Gram‐positive bacteria and 3–4 mm for Gram‐negative bacteria. The strongest antibacterial activity was observed against *Corynebacterium diphtheriae* with an inhibition zone of 8.67 ± 0.57 mm, while *Bacillus subtilis* showed 6 ± 0 mm inhibition. MIC values ranged from 62.5 to 500 mg mL^−1^, with *Staphylococcus aureus* being the most sensitive microorganism (MIC 62.5 mg mL^−1^; MBC 125 mg mL^−1^) and *Proteus mirabilis* the most resistant (MIC and MBC 500 mg mL^−1^). The antimicrobial effect was attributed to fatty acids and bioactive compounds from fish and lemon oils that disrupted bacterial membranes, increased membrane permeability, and induced bacteriostatic and bactericidal effects, while nanoemulsification enhanced dispersion and interaction with microbial cells.

The study evidences a model in which EPA and DHA exhibit antimicrobial effects by a combination of membrane disruption, interference with microbial energetics and homeostasis, and inhibition of biofilm formation. Omega 3 lipids add to the physiological benefits and suggest that their incorporation into functional foods, especially when stabilized via encapsulation to protect against oxidation, can provide both health benefits to the host and food safety by rendering contaminating or pathogenic microbes less viable and robust. However, the exact molecular targets and the relative contribution of each mechanism across different microbial actions still need to be elucidated.

### 7.2 Antimicrobial Functionality of Fish Oil‐Fortified Foods

Fish oil has been used as a food ingredient for its antimicrobial activity, and its major omega‐3 fatty acids, EPA and DHA, are known to interfere with microbial membrane integrity and crucial cellular processes. The experimental evidence shows that these polyunsaturated fatty acids integrate into bacterial lipid bilayers, increasing membrane permeability and ultimately impairing microbial viability. EPA or DHA reducing populations of oral pathogenic bacteria and causing observable structural damage to microbial cells in an in vitro multi‐species biofilm model confirmed by scanning electron microscopy, demonstrating that membrane destabilization is a key antimicrobial mechanism, suggesting that fish oil‐derived lipids may play a role in microbial control in functional food systems. However, their antimicrobial activity is highly species‐dependent, with Gram‐negative bacteria generally showing lower susceptibility than Gram‐positive organisms because of the additional permeability barrier of the outer membrane. Therefore, although EPA and DHA show measurable antimicrobial activity, the relatively high inhibitory concentrations required and the variation among microbial taxa indicate that improved delivery systems and combined antimicrobial approaches should be considered in designing strategies for using fish oil as a multi‐purpose ingredient in fortified foods (Ribeiro‐Vidal et al. 2020).

Other investigations of fish oil lipids have demonstrated that DHA and EPA can substantially inhibit the development of *Staphylococcus aureus* biofilm formation at concentrations of 20 μg mL^−1^ and reduce the hemolytic activity associated with bacterial pathogenicity. These fatty acids were shown to downregulate virulence determinants, such as the α‐hemolysin gene (hla), via molecular analyses, suggesting that omega‐3 lipids not only inhibit microbial proliferation but also interrupt toxin production and pathogenic signaling pathways (Kim et al. 2018). Other complementary antimicrobial assays have shown that the MICs of DHA generally fall in the range of approximately 25–100 μM, depending on the species of bacteria, and bactericidal activity is observed at concentrations of 100 μM, demonstrating that antimicrobial efficacy is highly concentration‐dependent and differs between microbial taxa (Ribeiro‐Vidal et al. 2020). These quantitative thresholds indicate that although fish oil–derived omega‐3 fatty acids possess measurable antimicrobial activity, the relatively high concentrations required for microbial inhibition may limit their direct application in fortified foods due to potential impacts on sensory quality and oxidative stability.

Omega 3 PUFAs are reported to have measurable antimicrobial effects against a variety of Gram‐positive and Gram‐negative bacteria (Simplice et al. 2018), which are moderate in magnitude and spectrum, context dependent, and often mechanistically indirect. Meta‐analyses and narrative reviews stress that PUFAs can disrupt bacterial cell membranes, alter surface hydrophobicity, interfere with enzyme systems, and reduce biofilm formation, thereby inhibiting growth and virulence in vitro. These effects often require relatively high concentrations and show variable efficacy depending on the organism and environmental conditions such as temperature or presence of biofilm matrices (Clare et al. 2024). For instance, EPA and DHA show considerable decreases in biofilm biomass or MIC/MBC values against susceptible strains, but not as effectively against encapsulated or multidrug‐resistant bacteria. Moreover, in vivo and food‐system studies suggest that antimicrobial outcomes are influenced by host factors, delivery matrices, and processing conditions, limiting broad clinical extrapolation.

## 8 Fish Oil Fortified Functional Foods

### 8.1 Fish Oil Fortified Functional Yogurt

The fortification of functional foods with fish‐oil‐derived omega‐3 lipids was a strategy to enhance dietary intake of long‐chain polyunsaturated fatty acids. However, studies demonstrate substantial variability in technological efficiency, oxidative stability, and consumer acceptability depending on the encapsulation system and food matrix. In the study by Yang et al. (2024) hollow solid lipid micro‐ and nanoparticles were used to fortify milk with omega‐3 fatty acids. The authors reported encapsulation efficiencies approaching 88%–92% and an increase in vitro bioaccessibility from approximately 42% for free fish oil to nearly 71% for the encapsulated system. While this resulted in better short‐term stability, peroxide values rose from 1.9 to nearly 6.5 meq O_2_/kg lipid after 4 weeks, suggesting that lipid oxidation was only partially inhibited by the hollow lipid carriers, which are not adequate for long‐term dairy applications due to the high oxygen permeability and light exposure of liquid milk matrices. Another drawback for industrial translation of the study was that sensory thresholds for fishy off‐flavors were not explored during simulated digestion.

Another nanotechnology‐based yogurt fortification system using nanoemulsified fish oil was performed. Yogurt with fish‐oil nanoemulsion with fruit matrices was developed with droplet sizes less than 200 nm and peroxide values reduced from 4.8 to about 2.1 meq O_2_/kg when fruit polyphenols were added as natural antioxidants. Sensory scores were enhanced from 5.6 to 7.8 on a nine‐point hedonic scale compared with non‐encapsulated oil, but the study showed that nanoemulsions alone are not sufficient to inhibit oxidative degradation during fermentation and refrigerated storage; thiobarbituric acid reactive substances increased by over 35% after 21 days, which suggests that the large surface area of nanoemulsion droplets may enhance lipid oxidation if antioxidant systems are inadequate (Shanuke et al. 2025).

Another study by Mahfoudhi et al. (2022) employed a complex coacervation system of almond gum and gelatin to encapsulate fish oil microcapsules at an encapsulation efficiency of 70.5% with an average particle size of approximately 3.2 μm. The encapsulated omega‐3 fatty acids in the fortified yogurt remained relatively stable throughout 4 weeks of storage, with a relative improvement of approximately 15% in the fortified yogurt containing encapsulated fish oil (retained about 82% of total omega‐3 fatty acids) as compared to the unencapsulated fish oil (retained about 67% of total omega‐3 fatty acids). However, the relatively large particle size of the coacervate capsules indicates limited dispersion within the yogurt matrix, which may impact mouthfeel and EPA and DHA release kinetics during digestion.

In yogurt fortified with fish‐oil nanoemulsions stabilized by surfactant systems, peroxide values after 21 days of storage were approximately 0.28 mmol L^−1^ compared with 0.65 mmol L^−1^ in yogurt containing unencapsulated fish oil, indicating more than a twofold reduction in lipid oxidation. Similarly, retention of EPA and DHA remained near 95% and 94% of their initial concentrations, while non‐encapsulated systems declined to approximately 72% and 53%, respectively. These quantitative differences confirm the protective capacity of nanoemulsion systems; however, they also show that oxidative degradation is not entirely inhibited, even under refrigerated storage, and that highly unsaturated fatty acids are particularly vulnerable in acidic fermented dairy matrices (Zhong et al. 2018).

Therefore, future research should aim to develop integrative food‐matrix engineering approaches that simultaneously optimize encapsulation efficiency, sensory quality, rheology, and bioavailability. Comparative studies evaluating nanoemulsions, solid lipid nanoparticles, cyclodextrin inclusion complexes, and multilayer biopolymer capsules under the same fermentation and storage conditions are particularly needed to set reliable performance benchmarks. Finally, clinical and simulated digestion studies quantifying the actual bioaccessibility of EPA and DHA from fortified yogurt remain essential to validate the functional food claims associated with omega‐3 dairy products.

### 8.2 Fish‐Oil Fortification in Bakery and Pasta

Fish‐oil fortification of bakery and pasta products is a scientifically proven method to improve the nutritional functionality of staple foods by increasing long‐chain omega‐3 polyunsaturated fatty acids (PUFAs), especially EPA and DHA, and the dietary omega‐6/omega‐3 ratio. Although scientific evidence shows that fish‐oil fortification can dramatically increase total PUFA content and reduce relative saturated lipid fractions, resulting in improved lipid quality indices linked to cardiovascular health. However, recent studies show that there are still challenges with fish‐oil‐fortified foods related to lipid stability, sensory acceptability, and functional dosage (Shrirang and Sharma 2023).

In a study by Uriho et al. (2024), bread was fortified with microencapsulated fish oil and vitamin C, with an encapsulation efficiency of 78%–82%, and EPA and DHA retained 85% after baking at 180°C for 20 min. Although this protection reduced the increase in peroxide values from 0.4 to 1.2 meq O_2_ kg^−1^ post‐baking (compared to 6.2–7.0 in non‐encapsulated controls), thermal stress still caused significant lipid oxidation. Sensory analysis revealed that odor and taste scores decreased when fish‐oil inclusion exceeded 1.5% (w/w), suggesting a trade‐off between nutritional enhancement and organoleptic quality. In a similar study, Damerau et al. (2022) reported that spray‐dried fish‐oil emulsions using maltodextrin, plant, and whey proteins retained 83%–87% EPA + DHA after 28 days at 20°C (vs. 65% in non‐encapsulated controls), but TOTOX values increased from 4 to 10, and off‐flavors were detected at ≥ 2% inclusion.

In pasta‐based fortification, fortification enhances omega‐3 retention and protein–lipid interactions, whereas in bakery matrices, structured delivery systems improve oxidative stability, preserve bioactivity during thermal processing, and maintain acceptable rheological and sensory properties. The durum wheat spaghetti fortified with *Portulaca oleracea* was found to contain 250 mg EPA + DHA per 100 g serving and retained 80% of the fatty acids after cooking (Melilli et al. 2020), but sensory analysis showed slight bitterness at higher fortification levels. Tilapia flour added to pasta supplied 300 mg EPA + DHA per 100 g, but lipid oxidation occurred during storage, with peroxide values rising from 0.35 to 1.1 meq O_2_ kg^−1^ over 30 days at 4°C (Monteiro et al. 2016), indicating that fish‐derived lipids in dried matrices are not fully stable.

In general, encapsulation and emulsion‐based delivery enhance retention of omega‐3 and reduce early‐stage oxidation but provide only 80%–95% protection, with peroxide or TOTOX values still increasing over storage or processing. The sensory limitation limits inclusion levels to 1%–2% w/w, which limits the functional dosage that can be achieved per serving. The need for integrated strategies that combine encapsulation, antioxidants, and matrix‐compatible delivery systems to achieve nutritionally meaningful doses of EPA and DHA at industrially scalable levels remains a critical gap in optimizing thermal stability and long‐term storage, as well as in developing sensory‐masked formulations.

### 8.3 Fish‐Oil Fortification in Meat Products

Fish‐oil fortification is a promising way to enhance the nutritional quality of meat products by adding long‐chain omega‐3 polyunsaturated fatty acids, especially eicosapentaenoic acid (EPA) and docosahexaenoic acid (DHA). Despite its benefits, the high susceptibility of fish oil to lipid oxidation is a major technological limitation affecting sensory quality, shelf stability, and consumer acceptability. Solomando et al. (2023) reported that incorporation of fish‐oil microcapsules (4.5%–6.5%) into meat products increased omega‐3 content sufficiently to meet nutritional claims with lower thiobarbituric acid reactive substances (TBARS) values in microencapsulated treatments compared with direct oil incorporation. Similarly, Kawecki et al. (2021) evaluated poultry sausages enriched with liquid and microencapsulated fish oil during refrigerated storage and found that microencapsulation markedly reduced oxidative deterioration. TBARS values in sausages containing liquid fish oil were slightly greater than microencapsulated treatments. The study also demonstrated enhanced retention of EPA and DHA in encapsulated systems.

Fish oil incorporation into meat matrices has been further improved using advanced delivery systems such as nanoemulsions, which enhanced lipid dispersion, oxidative resistance, and bioavailability because droplet sizes below 200 nm improved emulsion stability and reduced phase separation in processed meats (Ujilestari et al. 2023). In addition, nanoemulsion approaches can increase oxidative stability by 40%–60% compared with direct fortification (Jamshidi et al. 2020) and also mask undesirable fishy odors and flavors. Rodrigues et al. (2023) further highlighted that the replacement of saturated animal fat with fish‐oil‐based functional lipids reduced saturated fatty acid content and improved polyunsaturated/saturated fatty‐acid ratios in reformulated meat products, thereby supporting cardiovascular‐health‐oriented product development.

An alternative strategy for omega‐3 enrichment of meat products includes animal‐feeding strategies. The study, Komprda et al. (2021) showed that feeding pigs a diet containing 8% fish oil changed the fatty‐acid composition of sausages made from the pork. The result revealed increased EPA and DHA levels from undetectable to 360 mg/100 g in Vienna sausages and 214 mg/100 g in Bologna‐type salami, lowering the n‐6/n‐3 ratio from 13.9 to 2.8 and from 13.45 to 2.57, respectively. However, the oxidative stabilities TBARS values showed slightly increasing, without substantial sensory acceptances.

Fish‐oil fortification can significantly increase the EPA, DHA, and total omega‐3 fatty acid content in meat products. Advanced encapsulation technologies reduce lipid oxidation, enhance sensory acceptability, and increase bioaccessibility when compared with direct oil incorporation. However, oxidative deterioration is the main technical challenge in the fish‐oil fortification of meat products, especially during thermal processing and prolonged storage, and thus future research should be directed at optimizing encapsulation materials, antioxidant systems, and processing conditions to enhance the commercial viability and storage stability of omega‐3‐enriched functional meat products.

## 9 Knowledge Gaps and Future Research Directions

Valorization of fish processing waste into omega‐3 bioactive lipids was an important strategy to develop innovative functional foods. However, several critical limitations still hinder their effective incorporation into functional foods in large‐scale applications. The investigation of the physicochemical stability of fish oil‐enriched functional foods, along with the interactions between lipids and proteins or polysaccharides and the controlled release behavior within complex food matrices, remains limited. Additionally, the mechanistic understanding of the dual antioxidant and antimicrobial properties of EPA and DHA has not been thoroughly investigated. Identification of antioxidant and antimicrobial mechanisms of action of EPA and DHA and how they interact with bacterial cell membranes, oxidative radicals, and endogenous food components using membrane permeability assays, lipid oxidation kinetics, and molecular interaction should be determined. The synergistic combinations of omega‐3 lipids with natural preservatives, such as rosemary extract, thymol, eugenol, nisin, or phenolic compounds, should be explored to enhance microbial inhibition and lipid stabilization while minimizing the use of synthetic additives in fortified foods.

In food systems, the composition of the matrix, processing conditions, and gastrointestinal environments can affect stability, bioaccessibility, and biological efficacy. In particular, the interactions of nano‐ or microencapsulated fish oils with food proteins, polysaccharides, salts, and processing conditions such as heating, freezing, drying, and pH variation are minimally characterized. Furthermore, comparative studies evaluating different encapsulation technologies under identical storage and gastrointestinal conditions remain scarce, limiting the identification of the most effective delivery systems for functional food applications. Additionally, most release and digestion studies rely on static in vitro models, while dynamic gastrointestinal simulations and in vivo validations are rarely performed.

Future research should therefore emphasize integrative and scalable encapsulation approaches, including multilayered and stimuli‐responsive delivery systems, combined with mechanistic kinetic modeling and simulated gastrointestinal studies to validate functional performance. Moreover, techno‐economic assessment, multilayer matrix optimization, and life‐cycle analysis of fish‐waste‐derived omega‐3 ingredients are needed to develop sensory‐acceptable innovative products, industrial‐scale implementation, and sustainable circular bioeconomy strategies.

## 10 Conclusion

The conversion of fish processing waste into high‐value omega‐3 bioactive lipids is a sustainable and innovative strategy for functional food development. The main challenges of omega‐3 fatty acids were oxidation, off‐flavors, and low water solubility, which can limit direct application in food systems. This review demonstrates that encapsulation strategies, particularly nanoemulsions, nanoliposomes, and multilayer biopolymer systems, effectively improve omega‐3 oxidative stability, controlled release, bioaccessibility, and sensory quality by reducing fishy off‐flavors. Encapsulated omega‐3 lipids also provide dual function as antioxidants to reduce lipid peroxidation and as antimicrobials to disrupt microbial membranes and metabolism that can enhance food quality and shelf life of products. The review further highlights that omega‐3 encapsulation systems can improve microbial stability in fortified dairy, meat, and bakery products than free oil. However, the EPA and DHA industrial translation remains limited. Future research should concentrate on optimizing scalable encapsulation methods, exploring interactions in complex food matrices, and validating physiological efficacy in vivo. In general, valorization of fish waste with advanced encapsulation strategies provides a route for innovative, safe, and health‐promoting functional foods that can advance nutritional enrichment and circular bioeconomy of the seafood processing industry.

## Author Contributions

**Abebe Desalegn:** conceptualization, investigation, writing – original draft, visualization, validation, writing – review and editing, formal analysis. **Gadissa Mosisa:** conceptualization, investigation, writing – review and editing. **Molla Abeje:** conceptualization, investigation, writing – original draft, writing – review and editing, supervision. **Habtamu Admassu:** conceptualization, investigation, supervision, methodology, visualization, data curation, writing – review and editing. **Gebeyehu Ayele:** conceptualization, investigation, writing – review and editing. **Tanaji Kudre:** writing – review and editing, supervision, methodology.

## Funding

The authors have nothing to report.

## Ethics Statement

This study does not have an impact on animal or human health.

## Conflicts of Interest

The authors declare no conflicts of interest.

## Data Availability

No new data were generated or analyzed in this study. Data sharing is not applicable to this article as it is a review based on previously published literature.

## References

[fsn372178-bib-0001] Advaitha, S. , K. V. Sunooj , S. K. Vellaisamy , et al. 2026. “Valorization of Side Streams to Enhance Seafood Sustainability.” Sustainable Food Technology 4, no. 1: 89–109.

[fsn372178-bib-0002] Ahmad, I. , A. Dogra , T. Nagpal , et al. 2025. “Liposome‐Like Encapsulation of Fish Oil‐Based Self‐Nano Emulsifying Formulation for Improved Bioavailability.” Applied Food Research 5, no. 1: 100745.

[fsn372178-bib-0003] Ahmmed, M. K. , F. Ahmmed , I. Stewart , A. Carne , H. S. Tian , and A. E. D. A. Bekhit . 2021. “Omega‐3 Phospholipids in Pacific Blue Mackerel ( *Scomber australasicus* ) Processing By‐Products.” Food Chemistry 353: 129451. 33714118 10.1016/j.foodchem.2021.129451

[fsn372178-bib-0004] Alavi, S. E. , M. K. M. Esfahani , A. Raza , H. Adelnia , and H. E. Shahmabadi . 2022. “PEG‐Grafted Liposomes for Enhanced Antibacterial and Antibiotic Activities: An In Vivo Study.” NanoImpact 25: 100384. 35559890 10.1016/j.impact.2022.100384

[fsn372178-bib-0005] Aldhafiri, F. K. 2022. “Investigating the Role of EPA and DHA on Cellular Oxidative Stress; Profiling Antidiabetic and Antihypertensive Potential.” Journal of Pharmacy & Bioallied Sciences 14, no. 4: 178–185. 10.4103/jpbs.jpbs_383_22. 37051424 PMC10084997

[fsn372178-bib-0006] Amiri, H. , B. Shabanpour , and P. Pourashouri . 2023. “Encapsulation of Marine Bioactive Compounds Using Liposome Technique: Evaluation of Physicochemical Properties and Oxidative Stability During Storage.” Food Structure 35: 100308.

[fsn372178-bib-0007] Annamalai, A. , and R. Sasikumar . 2025. “Enzymatic Valorization of Fishery By‐Products: Harnessing Cold‐Adapted Marine Proteases for Sustainable Production of Bioactive Peptides.” Archives of Microbiology 207, no. 9: 232. 40833501 10.1007/s00203-025-04431-y

[fsn372178-bib-0008] Azmi, N. A. N. , A. A. M. Elgharbawy , H. M. Salleh , and M. Moniruzzaman . 2022. “Preparation, Characterization and Biological Activities of an Oil‐in‐Water Nanoemulsion From Fish By‐Products and Lemon Oil by Ultrasonication Method.” Molecules 27: 6725. 36235261 10.3390/molecules27196725 PMC9570546

[fsn372178-bib-0085] Caruso, G. , R. Floris , C. Serangeli , and L. Di Paola . 2020. “Fishery Wastes as a yet Undiscovered Treasure from the Sea: Biomolecules Sources, Extraction Methods and Valorization.” Marine Drugs 18, no. 12: 622. 33297310 10.3390/md18120622 PMC7762275

[fsn372178-bib-0010] Chen, L. , H. Gong , W. Zhang , et al. 2024. “Fish Oil‐Loaded Hydrogels Using a Dual Gelation Method of Fish Gelatin Cold‐Setting With Ion‐Alginate Cross‐Linking.” ACS Food Science & Technology 4, no. 10: 2517–2527.

[fsn372178-bib-0011] Chen, L. , W. Zhang , Q. Bian , X. Wang , and J. Zhong . 2025. “Controlled Preparation of Fish Oil Powders via Complex Coacervation and Genipin Crosslinking.” NPJ Science of Food 9, no. 1: 80. 40389434 10.1038/s41538-025-00448-1 PMC12089318

[fsn372178-bib-0084] Clare, J. , M. R. Lindley , and E. Ratcliffe . 2024. “The Antimicrobial and Antibiofilm Abilities of Fish oil Derived Polyunsaturated Fatty Acids and Manuka Honey.” Microorganisms 12, no. 4: 778. 38674722 10.3390/microorganisms12040778 PMC11052219

[fsn372178-bib-0012] Coppola, D. , C. Lauritano , F. Palma Esposito , G. Riccio , C. Rizzo , and D. de Pascale . 2021. “Fish Waste: From Problem to Valuable Resource.” Marine Drugs 19, no. 2: 116. 33669858 10.3390/md19020116 PMC7923225

[fsn372178-bib-0013] Coraça‐Huber, D. C. , S. Steixner , A. Wurm , and M. Nogler . 2021. “Antibacterial and Anti‐Biofilm Activity of Omega‐3 Polyunsaturated Fatty Acids Against Periprosthetic Joint Infections‐Isolated Multi‐Drug Resistant Strains.” Biomedicine 9, no. 4: 334. 10.3390/biomedicines9040334 PMC8065983 33810261

[fsn372178-bib-0014] Dabora, S. , B. Jiang , and K. S. S. Hlaing . 2025. “Encapsulation of Fish Oil in Pullulan/Sodium Caseinate Nanofibers: Fabrication, Characterization, and Oxidative Stability.” Foods 14, no. 21: 3677. 41227649 10.3390/foods14213677 PMC12607480

[fsn372178-bib-0015] Damerau, A. , S. A. Mustonen , D. Ogrodowska , et al. 2022. “Food Fortification Using Spray‐Dried Emulsions of Fish Oil Produced With Maltodextrin, Plant and Whey Proteins—Effect on Sensory Perception, Volatiles and Storage Stability.” Molecules 27, no. 11: 3553. 10.3390/molecules27113553. 35684490 PMC9182505

[fsn372178-bib-0016] Debbarma, A. , V. Kumar , P. K. Mishra , et al. 2023. “Valorisation of Basa Fish ( *Pangasius bocourti* ) Waste: Oil Extraction and Encapsulation.” International Journal of Food Science and Technology 58, no. 5: 2764–2771.

[fsn372178-bib-0017] Desalegn, A. , H. Admassu , G. Mosissa , G. Ayele , and M. Abeje . 2026. “Review on Fish Waste Valorization: Advanced Fish Oil Extraction and Chitosan Application for Fish Oil Nano‐Encapsulation Technologies.” Journal of Aquatic Food Product Technology 35: 282–308. 10.1080/10498850.2026.2665441.

[fsn372178-bib-0083] Dragoev, S. G. 2024. “Lipid Peroxidation in Muscle Foods: Impact on Quality, Safety and Human Health.” Food 13, no. 5: 797. 10.3390/foods13050797 PMC10930712 38472909

[fsn372178-bib-0018] Fiori, L. , M. Solana , P. Tosi , M. Manfrini , C. Strim , and G. Guella . 2012. “Lipid Profiles of Oil From Trout ( *Oncorhynchus mykiss* ) Heads, Spines and Viscera: Trout By‐Products as a Possible Source of Omega‐3 Lipids?” Food Chemistry 134, no. 2: 1088–1095. 23107732 10.1016/j.foodchem.2012.03.022

[fsn372178-bib-0019] Food and Agriculture Organization of the United Nations . 2013. Food Wastage Footprint: Impacts on Natural Resources: Summary Report. FAO. https://www.fao.org/4/i3347e/i3347e.pdf.

[fsn372178-bib-0020] García‐Moreno, P. J. , K. Stephansen , J. van der Kruijs , et al. 2016. “Encapsulation of Fish Oil in Nanofibers by Emulsion Electrospinning: Physical Characterization and Oxidative Stability.” Journal of Food Engineering 183: 39–49.

[fsn372178-bib-0082] Guo, Y. , Z. Zhang , Q. Zeng , P. Liang , and F. Shi . 2025. “Fabrication and Characterization of Fish oil Microcapsules in the Presence of Large Yellow Croaker roe Phospholipids.” Food Hydrocolloids: 112125.

[fsn372178-bib-0021] Honrado, A. , S. Rubio , J. A. Beltrán , and J. Calanche . 2022. “Fish By‐Product Valorization as Source of Bioactive Compounds for Food Enrichment: Characterization, Suitability and Shelf Life.” Foods 11, no. 22: 3656. 36429248 10.3390/foods11223656 PMC9689964

[fsn372178-bib-0022] Ibi, A. , C. Chang , Y. C. Kuo , et al. 2024. “Evaluation of the Metabolite Profile of Fish Oil Omega‐3 Fatty Acids (n‐3 FAs) in Micellar and Enteric‐Coated Forms—A Randomized, Cross‐Over Human Study.” Metabolites 14, no. 5: 265. 38786742 10.3390/metabo14050265 PMC11123365

[fsn372178-bib-0023] Islam, F. , F. Saeed , M. Afzaal , M. Hussain , A. Ikram , and M. A. Khalid . 2023. “Food Grade Nanoemulsions: Promising Delivery Systems for Functional Ingredients.” Journal of Food Science and Technology 60, no. 5: 1461–1471. 10.1007/s13197-022-05387-3. 37033316 PMC10076486

[fsn372178-bib-0025] Jamshidi, A. , H. Cao , J. Xiao , and J. Simal‐Gandara . 2020. “Advantages of Techniques to Fortify Food Products With the Benefits of Fish Oil.” Food Research International 137: 109353. 33233057 10.1016/j.foodres.2020.109353

[fsn372178-bib-0026] Jiang, M. , Z. Hu , X. D. Chen , and P. Wu . 2025. “Unlocking the Release, Digestion and Absorption Kinetics of DHA From Different Fish Oil Delivery Systems: An In Vitro and Ex Vivo Study.” Food Hydrocolloids 162: 110966.

[fsn372178-bib-0027] Jimenez‐Champi, D. , F. L. Romero‐Orejon , A. M. Muñoz , and F. Ramos‐Escudero . 2024. “The Revalorization of Fishery By‐Products: Types, Bioactive Compounds, and Food Applications.” International Journal of Food Science 2024, no. 1: 6624083. 39105167 10.1155/2024/6624083 PMC11300074

[fsn372178-bib-0028] Kalkan, E. , H. Keskin Çavdar , and M. Maskan . 2025. “Health Impacts and Innovative Extraction Methods of Fish Oil: A Review.” European Journal of Lipid Science and Technology 127, no. 2: e202400171.

[fsn372178-bib-0029] Kalladathvalappil Venugopalan, V. , L. Ramadevi Gopakumar , A. Kumaran Kizhakkeppurath , et al. 2024. “Enhancement of Oxidative Stability of Polyunsaturated Fatty Acid‐Rich Fish Oil: Microencapsulation Using Chitosan‐Whey Protein Complex and Betalain.” International Journal of Food Science and Technology 59, no. 4: 2286–2296.

[fsn372178-bib-0030] Kawecki, K. , J. Stangierski , and P. Konieczny . 2021. “An Analysis of Oxidative Changes and the Fatty Acid Profile in Stored Poultry Sausages With Liquid and Microencapsulated Fish Oil Additives.” Molecules 26, no. 14: 4293. 10.3390/molecules26144293. 34299569 PMC8303385

[fsn372178-bib-0031] Khoshnood, S. , B. Negahdari , V. H. Kaviar , et al. 2023. “Amoxicillin‐Docosahexaenoic Acid Encapsulated Chitosan‐Alginate Nanoparticles as a Delivery System With Enhanced Biocidal Activities Against *Helicobacter pylori* and Improved Ulcer Healing.” Frontiers in Microbiology 14: 1083330. 36846798 10.3389/fmicb.2023.1083330 PMC9948253

[fsn372178-bib-0032] Kim, Y. G. , J. H. Lee , C. J. Raorane , S. T. Oh , J. G. Park , and J. Lee . 2018. “Herring Oil and Omega Fatty Acids Inhibit *Staphylococcus aureus* Biofilm Formation and Virulence.” Frontiers in Microbiology 9: 1241. 10.3389/fmicb.2018.01241. 29963020 PMC6014104

[fsn372178-bib-0033] Komprda, T. , M. Jůzl , M. Matejovičová , et al. 2021. “Fatty Acid Composition, Oxidative Stability, and Sensory Evaluation of the Sausages Produced From the Meat of Pigs Fed a Diet Enriched With 8% of Fish Oil.” Journal of Food Science 86, no. 6: 2312–2326. 33963532 10.1111/1750-3841.15749

[fsn372178-bib-0034] Korma, S. A. , A. Rehman , A. Hussain , et al. 2025. “Valorization of Fish Oil From Processing By‐Products: Composition, Nutritional Value, and Potential Applications.” Applied Food Research 5: 101552.

[fsn372178-bib-0035] Kumar, P. , and S. Chauhan . 2025. “Advances in Fish Oil Extraction and Encapsulation Techniques: Enhancing Nutritional Value and Stability.” International Journal 14, no. 3: 1534–1538.

[fsn372178-bib-0080] Kumar, S. , V. Kumar , N. B. Rathod , and N. Nirmal . 2025. “Seafood Processing Waste and Protein Content.” In Fish Protein Hydrolysates, 1–30. Academic Press.

[fsn372178-bib-0037] Li, Y. , and G. Liu . 2024. “Encapsulation of Antarctic Krill Oil and Kaempferol Co‐Loaded Nano‐Liposomes in Alginate‐Chitosan Hydrogel Beads: Improved Stability and Modified Digestive Behaviour.” International Journal of Food Science and Technology 59, no. 5: 3198–3211.

[fsn372178-bib-0038] Liu, L. , L. Tao , J. Chen , et al. 2021. “Fish Oil‐Gelatin Core‐Shell Electrospun Nanofibrous Membranes as Promising Edible Films for the Encapsulation of Hydrophobic and Hydrophilic Nutrients.” LWT 146: 111500.

[fsn372178-bib-0039] Mahfoudhi, N. , M. Isseoui , L. Rezig , and R. Ksouri . 2022. “Enrichment of Yogurt With Encapsulated Fish Oil by Complex Coacervation Using Almond Gum and Gelatin as Wall Materials.” Journal of Aquatic Food Product Technology 31, no. 9: 883–898.

[fsn372178-bib-0040] Melilli, M. G. , A. Pagliaro , S. Scandurra , C. Gentile , and V. Di Stefano . 2020. “Omega‐3 Rich Foods: Durum Wheat Spaghetti Fortified With *Portulaca oleracea* .” Food Bioscience 37: 100730. 10.3390/foods9060764 PMC7353616 32531917

[fsn372178-bib-0041] Miralles‐Pérez, B. , L. Méndez , M. R. Nogués , et al. 2021. “Effects of a Fish Oil Rich in Docosahexaenoic Acid on Cardiometabolic Risk Factors and Oxidative Stress in Healthy Rats.” Marine Drugs 19, no. 10: 555. 10.3390/md19100555. 34677454 PMC8539050

[fsn372178-bib-0042] Monteiro, M. L. , E. T. Mársico , M. S. Soares Jr. , et al. 2016. “Nutritional Profile and Chemical Stability of Pasta Fortified With Tilapia ( *Oreochromis niloticus* ) Flour.” PLoS One 11, no. 12: e0168270. 10.1371/journal.pone.0168270. 27973565 PMC5156385

[fsn372178-bib-0043] Mude, M. G. 2024. “Different Gelatins Used in the Encapsulation of Fish Oil.” Asian Journal of Pharmaceutics (AJP) 18, no. 4: 1199.

[fsn372178-bib-0044] Naseem, S. , A. Imam , A. S. Rayadurga , A. Ray , and S. K. Suman . 2024. “Trends in Fisheries Waste Utilization: A Valuable Resource of Nutrients and Valorized Products for the Food Industry.” Critical Reviews in Food Science and Nutrition 64, no. 25: 9240–9260. 10.1080/10408398.2023.2211167. 37183680

[fsn372178-bib-0046] Obukhova, E. S. , and S. A. Murzina . 2024. “Mechanisms of the Antimicrobial Action of Fatty Acids (Review).” Applied Biochemistry and Microbiology 60, no. 6: 580–588. 10.31857/S0555109924060029.

[fsn372178-bib-0047] Okur, P. S. , D. Ciftci , and O. N. Ciftci . 2025. “Encapsulation of Fish Oil in Natural Wax‐Based Solid Lipid Particles Using Supercritical Carbon Dioxide: A Green Strategy to Develop Stable Powder Fish Oil.” Sustainable Food Technology 3, no. 2: 436–444.

[fsn372178-bib-0048] Paul, M. , S. G. Kang , J. Im , and W. J. Song . 2024. “Development of a Fish Oil–Nanoemulsion Gel as a Drug‐Delivery System to Prevent Capsular Contracture.” Scientific Reports 14, no. 1: 29385. 39592695 10.1038/s41598-024-81122-6 PMC11599770

[fsn372178-bib-0081] Peng, J. , W. Zhang , Y. Zi , et al. 2024. “Fish oil Encapsulation by Genipin‐Crosslinked Complex Coacervation Between Gelatin and Different Anionic Polysaccharides.” Food Hydrocolloids 152: 109945.

[fsn372178-bib-0049] Perez‐Palacios, T. , J. Ruiz‐Carrascal , J. C. Solomando , F. De‐la‐Haba , A. Pajuelo , and T. Antequera . 2022. “Recent Developments in the Microencapsulation of Fish Oil and Natural Extracts: Procedure, Quality Evaluation and Food Enrichment.” Foods 11, no. 20: 3291. 37431039 10.3390/foods11203291 PMC9601459

[fsn372178-bib-0050] Perumal, S. , R. Atchudan , and W. Lee . 2022. “A Review of Polymeric Micelles and Their Applications.” Polymers 14, no. 12: 2510. 35746086 10.3390/polym14122510 PMC9230755

[fsn372178-bib-0051] Purba, D. T. , J. Dave , and P. Somboon . 2025. “Sustainable Oleogel Encapsulation of Tilapia Fish Oil Using Protein‐Based Cryogels: Fabrication, Characterization, and Digestion Behavior.” Sustainable Food Technology 3, no. 5: 1480–1491.

[fsn372178-bib-0052] Rahim, M. A. , M. Uzma , F. Al‐Asmari , et al. 2025. “A Comprehensive Review on Nutritional Traits, Extraction Methods, Oxidative Stability, Encapsulation Technologies, Food Applications and Health Benefits of Omega Fatty Acids.” Food Science & Nutrition 13, no. 10: e71008. 41030839 10.1002/fsn3.71008 PMC12477334

[fsn372178-bib-0053] Rahmani‐Manglano, N. E. , E. Z. Fallahasghari , A. C. Mendes , et al. 2024. “Oxidative Stability of Fish Oil‐Loaded Nanocapsules Produced by Electrospraying Using Kafirin or Zein Proteins as Wall Materials.” Antioxidants 13, no. 9: 1145. 39334804 10.3390/antiox13091145 PMC11428463

[fsn372178-bib-0054] Ribeiro‐Vidal, H. , M. C. Sánchez , A. Alonso‐Español , et al. 2020. “Antimicrobial Activity of EPA and DHA Against Oral Pathogenic Bacteria Using an In Vitro Multi‐Species Subgingival Biofilm Model.” Nutrients 12, no. 9: 2812. 32937742 10.3390/nu12092812 PMC7551721

[fsn372178-bib-0055] Rodrigues, S. S. Q. , L. Vasconcelos , A. Leite , I. Ferreira , E. Pereira , and A. Teixeira . 2023. “Novel Approaches to Improve Meat Products' Healthy Characteristics: A Review on Lipids, Salts, and Nitrites.” Foods 12, no. 15: 2962. 10.3390/foods12152962. 37569231 PMC10418592

[fsn372178-bib-0056] Sakai, C. , M. Ishida , H. Ohba , et al. 2017. “Fish Oil Omega‐3 Polyunsaturated Fatty Acids Attenuate Oxidative Stress‐Induced DNA Damage in Vascular Endothelial Cells.” PLoS One 12, no. 11: e0187934. 10.1371/journal.pone.0187934. 29121093 PMC5679535

[fsn372178-bib-0057] Saw, C. L. , A. Y. Yang , Y. Guo , and A. N. Kong . 2013. “Astaxanthin and Omega‐3 Fatty Acids Individually and in Combination Protect Against Oxidative Stress via the Nrf2‐ARE Pathway.” Food and Chemical Toxicology: An International Journal Published for the British Industrial Biological Research Association 62: 869–875. 10.1016/j.fct.2013.10.023. 24157545

[fsn372178-bib-0058] Shahidi, F. , and P. Ambigaipalan . 2018. “Omega‐3 Polyunsaturated Fatty Acids and Their Health Benefits.” Annual Review of Food Science and Technology 9: 345–381. 10.1146/annurev-food-111317-095850 29350557

[fsn372178-bib-0059] Shanuke, D. S. , E. M. R. K. B. Edirisinghe , R. A. U. J. Marapana , and S. Hettiarachi . 2025. “Development of Omega‐3 Fortified Stirred Yoghurt With Enhanced Sensory and Oxidative Qualities Through the Addition of Fish Oil Nanoemulsion and Fruits.” International Journal of Food Science and Technology 60, no. 1: vvae034.

[fsn372178-bib-0060] Shehzad, Q. , A. Rehman , S. M. Jafari , et al. 2021. “Improving the Oxidative Stability of Fish Oil Nanoemulsions by Co‐Encapsulation With Curcumin and Resveratrol.” Colloids and Surfaces B: Biointerfaces 199: 111481. 33360079 10.1016/j.colsurfb.2020.111481

[fsn372178-bib-0061] Shrirang, P. V. , and M. Sharma . 2023. “Functional Aspects of Foods Fortified With Omega‐3 Fatty Acids.” In Plant‐Based Bioactive Compounds and Food Ingredients: Encapsulation, Functional, and Safety Aspects, edited by J. A. Malik , M. R. Goyal , P. Birwal , and R. B. Watharkar , 73–99. Apple Academic Press. 10.1201/9781003372226-5.

[fsn372178-bib-0062] Simplice, M. R. , W. H. Macaire , N. N. F. Hervé , et al. 2018. “Chemical Composition and Antibacterial Activity of Oils From Chrysicthys Nigrodigitatus and *Hepsetus odoe* , Two Freshwater Fishes From Yabassi, Cameroon.” Lipids in Health and Disease 17, no. 1: 45. 29530030 10.1186/s12944-018-0690-z PMC5848570

[fsn372178-bib-0063] Solomando, J. C. , F. Vazquez , T. Antequera , C. Folgado , and T. Perez‐Palacios . 2023. “Addition of Fish Oil Microcapsules to Meat Products–Implications for Omega‐3 Enrichment and Salt Reduction.” Journal of Functional Foods 105: 105575.

[fsn372178-bib-0064] Strobel, S. A. , K. Hudnall , B. Arbaugh , J. C. Cunniffe , H. B. Scher , and T. Jeoh . 2020. “Stability of Fish Oil in Calcium Alginate Microcapsules Cross‐Linked by In Situ Internal Gelation During Spray Drying.” Food and Bioprocess Technology 13, no. 2: 275–287.

[fsn372178-bib-0066] Swaidan, A. , F. Grasso , F. Falco , F. Turrini , and R. Boggia . 2026. “Advances in Fish Oil Extraction and Enrichment: A Comprehensive Review of Conventional and Green Technologies for High‐Quality Omega‐3‐Rich Oil Production.” Sustainable Food Technology 4, no. 4: 3467–3492.

[fsn372178-bib-0067] Tang, W. , S. Pang , Y. Luo , Q. Sun , Q. Tian , and C. Pu . 2022. “Improved Protective and Controlled Releasing Effect of Fish Oil Microcapsules With Rice Bran Protein Fibrils and Xanthan Gum as Wall Materials.” Food & Function 13, no. 8: 4734–4747. 10.1039/d1fo03500b. 35388381

[fsn372178-bib-0068] Ujilestari, T. , A. Febrisiantosa , M. M. Sholikin , R. Wahyuningsih , and T. Wahyono . 2023. “Nanoemulsion Application in Meat Product and Its Functionality: Review.” Journal of Animal Science and Technology 65, no. 2: 275–292. 10.5187/jast.2022.e120. 37093896 PMC10119454

[fsn372178-bib-0069] Uriho, A. , K. Chen , F. Zhou , et al. 2024. “Functional Breads With Encapsulated Vitamin C and Fish Oil: Nutritional, Technological, and Sensory Attributes.” Antioxidants 13, no. 11: 1325. 10.3390/antiox13111325. 39594466 PMC11590905

[fsn372178-bib-0070] Venugopalan, V. K. , L. R. Gopakumar , A. K. Kumaran , et al. 2021. “Encapsulation and Protection of Omega‐3‐Rich Fish Oils Using Food‐Grade Delivery Systems.” Foods 10, no. 7: 1566. 10.3390/foods10071566. 34359436 PMC8305697

[fsn372178-bib-0071] Wang, S. , H. Bai , T. Liu , J. Yang , and Z. Wang . 2024. “Optimization of Concentrations of Different n‐3PUFAs on Antioxidant Capacity in Mouse Hepatocytes.” Lipids in Health and Disease 23, no. 1: 214. 38982376 10.1186/s12944-024-02202-0 PMC11232338

[fsn372178-bib-0072] Waqar, M. , N. Sajjad , Q. Ullah , et al. 2025. “Fish By‐Products Utilization in Food and Health: Extraction Technologies, Bioactive, and Sustainability Challenges.” Food Science & Nutrition 13, no. 11: e71184. 41234609 10.1002/fsn3.71184 PMC12605986

[fsn372178-bib-0073] Xia, F. L. W. , S. Supri , H. Djamaludin , et al. 2024. “Turning Waste Into Value: Extraction and Effective Valorization Strategies of Seafood By‐Products.” Waste Management Bulletin 2, no. 3: 84–100.

[fsn372178-bib-0074] Xie, P. , Z. Tang , B. Guan , et al. 2025. “Preparation of Fish Oil Microcapsules for Enhanced Stability and Controlled Release via Plug‐and‐Play Microfluidic Chips.” Food and Bioproducts Processing 154: 216–228.

[fsn372178-bib-0075] Yang, H. , P. Wen , K. Feng , M. H. Zong , W. Y. Lou , and H. Wu . 2017. “Encapsulation of Fish Oil in a Coaxial Electrospun Nanofibrous Mat and Its Properties.” RSC Advances 7, no. 24: 14939–14946.

[fsn372178-bib-0076] Yang, J. , D. Ciftci , and O. N. Ciftci . 2024. “Fortification of Milk With Omega‐3 Fatty Acids Using Novel Bioactive‐Carrier Hollow Solid Lipid Micro‐ and Nanoparticles for Improved Omega‐3 Stability and Bioaccessibility.” ACS Food Science & Technology 4, no. 4: 813–820.

[fsn372178-bib-0077] Zhang, Q. , H. Zhu , R. Wang , et al. 2025. “Multi‐Modified β‐Lg‐Stabilized Fish Oil Emulsions: Physicochemical Stability, Antioxidant Capacity, and Bioavailability in Caco‐2 Cells.” Food Chemistry 497: 147064. 41232331 10.1016/j.foodchem.2025.147064

[fsn372178-bib-0078] Zhong, J. , R. Yang , X. Cao , X. Liu , and X. Qin . 2018. “Improved Physicochemical Properties of Yogurt Fortified With Fish Oil/γ‐Oryzanol by Nanoemulsion Technology.” Molecules 23, no. 1: 56. 10.3390/molecules23010056. 29301277 PMC6017217

[fsn372178-bib-0079] Zhou, L. , M. Yuan , Y. Han , et al. 2025. “Micellar Casein Were Constructed to Improve the Encapsulation Efficiency of Algae Oil Docosahexaenoic Acid by Transglutaminase‐Coupled Phosphoserine Peptide Chelating With Ca^2+^ .” International Journal of Biological Macromolecules 297: 139939. 39824426 10.1016/j.ijbiomac.2025.139939

